# Non-invasive detection of coronary inflammation using computed tomography and prediction of residual cardiovascular risk (the CRISP CT study): a post-hoc analysis of prospective outcome data

**DOI:** 10.1016/S0140-6736(18)31114-0

**Published:** 2018-09-15

**Authors:** Evangelos K Oikonomou, Mohamed Marwan, Milind Y Desai, Jennifer Mancio, Alaa Alashi, Erika Hutt Centeno, Sheena Thomas, Laura Herdman, Christos P Kotanidis, Katharine E Thomas, Brian P Griffin, Scott D Flamm, Alexios S Antonopoulos, Cheerag Shirodaria, Nikant Sabharwal, John Deanfield, Stefan Neubauer, Jemma C Hopewell, Keith M Channon, Stephan Achenbach, Charalambos Antoniades

**Affiliations:** a Division of Cardiovascular Medicine, Radcliffe Department of Medicine, University of Oxford, Oxford, UK; b Department of Cardiology, Friedrich-Alexander-Universität Erlangen-Nürnberg, Erlangen, Germany; c Cleveland Clinic Heart and Vascular Institute, Cleveland, OH, USA; d Cardiology Department, Oxford University Hospitals NHS Foundation Trust, Oxford, UK; e University College London Institute of Cardiovascular Science, London, UK; f Oxford Centre of Research Excellence, British Heart Foundation, Oxford, UK; g Oxford Biomedical Research Centre, National Institute of Health Research, Oxford, UK; h Clinical Trial Service Unit, Nuffield Department of Population Health, University of Oxford, Oxford, UK; i Caristo Diagnostics, Oxford, UK

## Abstract

**Background:**

Coronary artery inflammation inhibits adipogenesis in adjacent perivascular fat. A novel imaging biomarker—the perivascular fat attenuation index (FAI)—captures coronary inflammation by mapping spatial changes of perivascular fat attenuation on coronary computed tomography angiography (CTA). However, the ability of the perivascular FAI to predict clinical outcomes is unknown.

**Methods:**

In the Cardiovascular RISk Prediction using Computed Tomography (CRISP-CT) study, we did a post-hoc analysis of outcome data gathered prospectively from two independent cohorts of consecutive patients undergoing coronary CTA in Erlangen, Germany (derivation cohort) and Cleveland, OH, USA (validation cohort). Perivascular fat attenuation mapping was done around the three major coronary arteries—the proximal right coronary artery, the left anterior descending artery, and the left circumflex artery. We assessed the prognostic value of perivascular fat attenuation mapping for all-cause and cardiac mortality in Cox regression models, adjusted for age, sex, cardiovascular risk factors, tube voltage, modified Duke coronary artery disease index, and number of coronary CTA-derived high-risk plaque features.

**Findings:**

Between 2005 and 2009, 1872 participants in the derivation cohort underwent coronary CTA (median age 62 years [range 17–89]). Between 2008 and 2016, 2040 patients in the validation cohort had coronary CTA (median age 53 years [range 19–87]). Median follow-up was 72 months (range 51–109) in the derivation cohort and 54 months (range 4–105) in the validation cohort. In both cohorts, high perivascular FAI values around the proximal right coronary artery and left anterior descending artery (but not around the left circumflex artery) were predictive of all-cause and cardiac mortality and correlated strongly with each other. Therefore, the perivascular FAI measured around the right coronary artery was used as a representative biomarker of global coronary inflammation (for prediction of cardiac mortality, hazard ratio [HR] 2·15, 95% CI 1·33–3·48; p=0·0017 in the derivation cohort, and 2·06, 1·50–2·83; p<0·0001 in the validation cohort). The optimum cutoff for the perivascular FAI, above which there is a steep increase in cardiac mortality, was ascertained as −70·1 Hounsfield units (HU) or higher in the derivation cohort (HR 9·04, 95% CI 3·35–24·40; p<0·0001 for cardiac mortality; 2·55, 1·65–3·92; p<0·0001 for all-cause mortality). This cutoff was confirmed in the validation cohort (HR 5·62, 95% CI 2·90–10·88; p<0·0001 for cardiac mortality; 3·69, 2·26–6·02; p<0·0001 for all-cause mortality). Perivascular FAI improved risk discrimination in both cohorts, leading to significant reclassification for all-cause and cardiac mortality.

**Interpretation:**

The perivascular FAI enhances cardiac risk prediction and restratification over and above current state-of-the-art assessment in coronary CTA by providing a quantitative measure of coronary inflammation. High perivascular FAI values (cutoff ≥–70·1 HU) are an indicator of increased cardiac mortality and, therefore, could guide early targeted primary prevention and intensive secondary prevention in patients.

**Funding:**

British Heart Foundation, and the National Institute of Health Research Oxford Biomedical Research Centre.

## Introduction

Coronary computed tomography angiography (CTA) is a sensitive and widely used non-invasive imaging modality for diagnosing coronary artery disease;^1^, ^2^ it is also used for diagnosis and management of chest pain.^3^ However, coronary CTA focuses predominantly on identification of anatomically significant coronary artery stenosis, which is seen in fewer than 50% of patients referred for this test.^1^, ^2^ Importantly, most acute coronary syndromes are caused by unstable but non-obstructive atherosclerotic plaques,^4^ which cannot be identified by current non-invasive diagnostic tests detecting coronary luminal stenosis or stress-induced myocardial ischaemia.^5^, ^6^ Vascular inflammation is a driver of coronary atherosclerotic plaque formation and is a typical feature of atherosclerotic plaque rupture, leading to acute coronary syndrome.^7^ Currently, no method is readily available to allow early detection of vascular inflammation in coronary arteries. Such a method would enable timely deployment of measures to prevent disease development and future heart attacks.

**Research in context**

**Evidence before this study**

We searched PubMed, Scopus, Web of Science, and Embase for studies published before December, 2017, with the terms: (“epicardial adipose tissue”, “epicardial fat”, “subepicardial adipose tissue”, “subepicardial fat”, “cardiac adipose tissue”, “cardiac fat”, “pericoronary adipose tissue”, “pericoronary fat”, OR “perivascular fat”) AND (“coronary artery disease”, “coronary artery calcification”, “coronary artery calcium score”, “coronary stenosis”, “coronary atherosclerosis”, “myocardial ischemia”, “myocardial perfusion defects”, “acute coronary syndrome”, “major adverse cardiovascular events”, OR “cardiovascular disease”) AND (“computed tomography”, “radiodensity”, “density”, OR “attenuation”). We restricted our search to studies published in English. Of 910 articles retrieved, we identified no cohort studies investigating the prognostic value of perivascular fat attenuation phenotyping on coronary computed tomography angiography (CTA). Perivascular fat attenuation was first described in translational work, in which mechanistic proof-of-concept studies validated the hypothesis that coronary inflammation results in impaired lipid accumulation and adipocyte differentiation in adjacent perivascular fat. As a result, perivascular fat reduces its lipid content in response to inflammatory signals it receives from the adjacent coronary artery and provides a so-called sensor of coronary inflammation. We developed an imaging biomarker—the perivascular fat attenuation index (FAI)—to visualise and quantify these changes in perivascular fat, by mapping spatial changes in perivascular fat attenuation using routine coronary CTA. Clinical studies also showed a significant association between coronary artery disease and higher perivascular FAI values, and dynamic changes of the perivascular FAI around culprit—but not stable—lesions in patients with acute coronary syndromes. Cross-sectional observational studies have also confirmed that high perivascular fat attenuation is linked to the presence of coronary atherosclerotic plaques—validated against intravascular ultrasound—and ruptured coronary lesions in acute myocardial infarction and spontaneous coronary artery dissection. Nevertheless, the added prognostic value of perivascular fat attenuation phenotyping in real-life cohorts of patients undergoing diagnostic coronary CTA remains unknown.

**Added value of this study**

Our study is the first to show the independent and incremental value of quantifying the perivascular FAI in real-life cohorts of patients undergoing diagnostic coronary CTA. High perivascular FAI values around the right coronary artery identified individuals at risk for all-cause and cardiac mortality, over and above established cardiovascular risk factors and current state-of-the-art coronary CTA interpretation methods, including coronary calcium, coronary artery disease extent, and high-risk plaque features. We used two independent and substantially different cohorts to identify and validate a perivascular FAI cutoff of −70·1 Hounsfield units or higher, which flags high-risk individuals with a fivefold to ninefold higher adjusted risk for cardiac death. These associations remained robust after appropriate sensitivity and subgroup analyses, according to presence or extent of disease and treatment after coronary CTA. Most importantly, the perivascular FAI significantly improved mortality risk discrimination and reclassification in both study populations beyond current prognostic risk models.

**Implications of all the available evidence**

Our study validates the prognostic role of the perivascular FAI, the first non-invasive biomarker of coronary inflammation measured by traditional coronary CTA, over and above the presence of anatomically significant coronary stenosis or calcification. Most coronary CTA scans done worldwide do not record any relevant coronary atherosclerosis, yet half of heart attacks happen without substantial coronary stenosis (because of rupture of minor but highly inflamed or unstable atherosclerotic plaques). The perivascular FAI identifies these individuals and might guide early deployment of targeted intensive measures of primary prevention. Since the perivascular FAI also identifies vulnerable plaques in patients with established coronary atherosclerosis (despite optimum treatment), this biomarker could guide targeted deployment of intensive (and typically expensive) measures of secondary prevention. For example, patients with known coronary artery disease already receiving optimum treatment (eg, antiplatelet and statin therapy) who present with abnormally high perivascular fat attenuation could be candidates for treatment with new anti-inflammatory agents (eg, canakinumab) or PCSK9 inhibitors to target their residual cardiovascular and inflammatory risk.

Signals released from the inflamed coronary artery diffuse to the perivascular adipose tissue, inhibiting local adipogenesis.^8^ This changes the composition of perivascular fat around inflamed arteries, shifting its attenuation on coronary CTA from the lipid (more negative Hounsfield unit [HU] values [eg, closer to −190 HU]) to the aqueous phase (less negative HU values [eg, closer to −30 HU]).^8^ We have developed an imaging biomarker, the perivascular fat attenuation index (FAI), that captures these inflammation-induced changes in perivascular fat attenuation, enabling early detection of coronary inflammation using routine coronary CTA.^8^ Although the perivascular FAI has shown the ability to detect coronary inflammation, its importance for clinical risk stratification is so far unknown.

We postulated that by quantifying coronary artery inflammation, the perivascular FAI could predict future adverse events, independent of the degree of coronary stenosis or other factors included in modern risk stratification of individuals undergoing coronary CTA, thus identifying high-risk patients who might benefit from more intensive therapeutic strategies.

## Methods

### Study design and participants

In the Cardiovascular RISk Prediction using Computed Tomography (CRISP-CT) study, we did a post-hoc analysis of outcome data gathered prospectively from two independent cohorts. The first cohort (derivation cohort) consisted of consecutive patients who underwent clinically indicated coronary CTA at Erlangen University Hospital, Erlangen, Germany. A subgroup of patients underwent further non-contrast cardiac CT to assess the extent of coronary calcification by the Agatston coronary calcium score (CCS), in line with local clinical practice.^9^, ^10^, ^11^, ^12^ The second cohort (validation cohort) consisted of consecutive patients who underwent coronary CTA at Cleveland Clinic, Cleveland, OH, USA. To create a study population reflective of the real-life patient population undergoing diagnostic coronary CTA, we included all consecutive patients (aged 16 years or older) in the study, unless they were referred specifically for assessment of congenital heart disease.

The research protocol was approved by all local institutional review boards, including material and data sharing agreements, with waiver of individual informed consent.

### Procedures

The primary objective was to assess the predictive value of the perivascular FAI for the two primary endpoints of all-cause mortality and cardiac mortality. Details of clinical data collection, definition of risk factors and study endpoints, event adjudication, and data analysis are in the appendix.

Indications for coronary CTA and presenting symptoms are summarised in the appendix. In both the derivation and validation cohorts, most patients had a low-to-mid pretest probability of coronary artery disease (median 30·4% [IQR 15·4–50·4] in the derivation cohort *vs* 21·5% [9·3–38·7] in the validation cohort; p<0·0001). Coronary CTA protocols and image analysis methods are described in the appendix.

All images were anonymised locally and transferred to a core laboratory (Academic Cardiovascular CT Unit, University of Oxford, Oxford, UK) for analysis on a dedicated workstation (Aquarius Workstation version 4.4.11-13; TeraRecon, Foster City, CA, USA) by investigators who were unaware of population demographics and outcomes. All scans were reviewed initially for quality and presence of artifacts precluding a reliable qualitative and quantitative evaluation. Low-quality scans were reviewed by at least two independent investigators before being excluded from subsequent analysis. We defined mild, moderate, and severe coronary stenoses as luminal stenosis of 25–49%, 50–69%, and 70% or larger, respectively.^13^ We defined obstructive coronary artery disease as the presence of one or more coronary lesions causing luminal stenosis of 50% or larger, and we assessed the extent of coronary artery disease using the modified Duke coronary artery disease index.^14^ We defined high-risk plaque features (spotty calcification, low-attenuation plaque, positive remodelling, and napkin-ring sign) as described previously (appendix).^15^ We measured the total epicardial adipose tissue volume in a semi-automated manner by tracking the contour of the pericardium from the level of the pulmonary artery bifurcation to the apex of the heart at the most caudal end.

To measure the perivascular FAI, we traced the proximal 40-mm segments of all three major epicardial coronary vessels (right coronary artery, left anterior descending artery, and left circumflex artery) and defined respective perivascular fat as the adipose tissue within a radial distance from the outer vessel wall equal to the diameter of the vessel, as described and validated previously.^8^ To avoid the effects of the aortic wall, we excluded the most proximal 10 mm of the right coronary artery and analysed the proximal 10–50 mm of the vessel, as described previously.^8^ In the left anterior descending artery and left circumflex artery, we analysed the proximal 40 mm of each vessel. We did not analyse the left main coronary artery because it is of variable length. We ascertained the perivascular FAI by quantifying the weighted perivascular fat attenuation after adjustment for technical parameters, based on the attenuation histogram of perivascular fat within the range −190 HU to −30 HU, as described previously (appendix).^8^ Similar to findings of previous validation studies,^8^ technical (tube voltage, lumen attenuation) and anatomical (vessel diameter) parameters were all associated weakly with perivascular fat attenuation, accounting for roughly 5% of its variation (R^2^=0·05, p<0·0001 in a multivariable model [appendix]). Intraobserver and interobserver agreement for the perivascular FAI was very good (intraclass correlation coefficient 0·987 [p<0·0001] and 0·980 [p<0·0001], respectively).

### Statistical analysis

We present continuous variables as mean (SD) or median (range), as appropriate. We compared categorical variables between two or more groups with the χ^2^ test, and we compared continuous variables between groups with either Student's *t* test or Mann-Whitney *U* test (for two groups, as appropriate) or by ANOVA (for three groups). We tested correlations between continuous variables with either Pearson's *r* (including the coefficient of determination R^2^) or Spearman's rank correlation coefficient, as appropriate. We tested the association between the perivascular FAI and technical or anatomical variables in multivariable linear regression models. If information for categorical risk factors was missing, we created a third group (yes, no, unknown [maximum degree of missingness 13·9%]). We assessed the prognostic value of the perivascular FAI for all-cause, cardiac, or non-cardiac mortality using multivariable Cox regression models, and we plotted unadjusted Kaplan-Meier curves. We log-transformed CCS before inclusion in regression models (ln[CCS + 1]). We used fractional polynomials to model non-linear relations of the perivascular FAI with time-to-mortality in Cox regression models, and we present graphically the best-fitting fractional polynomial models. We selected the optimum cutoff for the perivascular FAI by identifying the value that maximised Youden's *J* statistic (sum of sensitivity and specificity) on time-dependent receiver operating characteristic (ROC) curve analysis for cardiac mortality to ensure an optimum balance between sensitivity and specificity in our models. We then tested the prognostic value of the perivascular FAI (as a dichotomous variable) by multivariable Cox regression analysis after adjustment for age, sex, hypertension, hypercholesterolaemia, diabetes mellitus, smoking, epicardial obesity (measured as total epicardial adipose tissue volume), tube voltage, modified Duke coronary artery disease index, and number of high-risk plaque features. By adding the perivascular FAI in a baseline model consisting of traditional risk factors, we assessed the improvement in model performance and discrimination and risk classification by (1) comparing the *C* statistic (area under the curve [AUC]) of the two nested models (from time-dependent ROC analysis), (2) doing a likelihood ratio test (in Cox regression models), and (3) calculating the integrated discrimination improvement (IDI) and net reclassification improvement (NRI) indices for censored data. We used bootstrapping with 200 replications to calculate 95% CIs for AUC, NRI, and IDI.^16^ All models were independently tested in the two cohorts of the study. We assessed heterogeneity in further subgroup analyses with the *I*^2^ statistic. We assessed the performance of the perivascular FAI for both primary and secondary prevention in subgroup analyses done according to the extent of coronary calcification (measured as CCS on non-contrast CT scans in the derivation cohort only),^11^, ^17^ presence of obstructive coronary artery disease, the extent of coronary artery disease (assessed by the modified Duke coronary artery disease index),^14^ and presence of high-risk plaque features on coronary CTA.^15^

We did statistical analyses with Stata version 14.0 (Stata Corp, College Station, TX, USA) and R version 3.4.0 (*survival, survIDINRI, timeROC, survminer*, and *nricens*). All tests were two-sided and α was set at 0·05.

### Role of the funding source

The funders had no role in study design, data collection, data analysis, data interpretation, or writing of the report. EKO and CA had full access to all data in the study and take full responsibility for the integrity of the data and the accuracy of the data analysis. The corresponding author had final responsibility for the decision to submit for publication.

## Results

Between 2005 and 2009, 1993 consecutive patients were enrolled to the Erlangen study (derivation cohort) and underwent coronary CTA. 121 scans (6%) were excluded from the analysis because of technical considerations (appendix), therefore 1872 patients contributed outcome data to our study (table 1). 1178 (63%) participants in the derivation cohort were men and the median age of all patients was 62 years (range 17–89). Median follow-up after coronary CTA was 72 months (range 51–109) in the derivation cohort. 1415 patients also underwent non-contrast cardiac CTA to assess coronary calcification.

**Table 1:** Cohort demographics and clinical characteristics

		**Derivation cohort (Erlangen)**	**Validation cohort (Cleveland)**	**p value**
**Demographics**				
Patients in original cohort		1993	2246	..
Eligible patients included in study		1872	2040	..
Age (years)		62 (17–89)	53 (19–87)	<0·0001
Men		1178 (63%)	1126 (55%)	<0·0001
Risk factors*				
Hypertension		1068 (62%)	949 (47%)	<0·0001
Hypercholesterolaemia		930 (55%)	1126 (55%)	0·78
Diabetes mellitus		215 (12%)	219 (11%)	0·11
Smoking		221 (13%)	465 (23%)	<0·0001
**Drug at baseline**†				
Antiplatelet (aspirin, clopidogrel, or ticagrelor)		606 (38%)	987 (48%)	<0·0001
Statin		557 (35%)	813 (40%)	0·0011
ACEi or ARB		696 (43%)	599 (29%)	<0·0001
β-blocker		721 (45%)	303 (15%)	<0·0001
**Cardiac CT**				
CT scanner type				<0·0001
	2 × 64 Definition Flash (Siemens Healthcare)	1482 (79%)	..	..
	1 × 64 Sensation 64 (Siemens Healthcare)	339 (18%)	..	..
	2 × 128 Definition Flash (Siemens Healthcare)	71 (3%)	221 (11%)	..
	1 × 256 Brilliance iCT (Philips Healthcare)	..	1777 (87%)	..
	2 × 192 Somatom Force (Siemens Healthcare)	..	42 (2%)	..
Tube voltage				<0·0001
	100 kVp	415 (22%)	673 (33%)	..
	120 kVp	1457 (78%)	1367 (67%)	..
Total coronary calcium score				..
	<300	1153 (62%)	N/A	
	≥300	262 (14%)	N/A	
	N/A‡	457 (24%)	N/A	
**Maximum stenosis**				
None to mild (<25%)		673 (36%)	1033 (51%)	<0·0001
Mild (25–49%)		732 (39%)	721 (35%)	..
Moderate (50–69%)		226 (12%)	196 (10%)	..
Severe (≥70%)		241 (13%)	90 (4%)	..
**Prospective follow-up**				
Duration (months)		72 (51–109)	54 (4–105)	<0·0001
All-cause mortality		114 (6%)	85 (4%)	..
Confirmed cardiac mortality		26 (1%)	48 (2%)	..
Confirmed non-cardiac mortality		72 (4%)	35 (2%)	..

Data are median (range) or number (%). Some denominators differ because of missing data. p values are for the comparisons between cohorts and are derived from the Mann-Whitney *U* test (continuous variables) and Pearson's χ^2^ test (categorical variables). ACEi=angiotensin-converting-enzyme inhibitor. ARB=angiotensin-II-receptor blocker. CT=computed tomography. N/A=not available.

* 9·2% maximum missingness in derivation cohort.

† 13·9% maximum missingness in derivation cohort.

‡ In the derivation cohort, the coronary calcium score was not measured in patients who had no clinical indication.

Between 2008 and 2016, 2246 consecutive patients were enrolled to the Cleveland study (validation cohort) and underwent coronary CTA. 206 (9%) scans were excluded from the analysis; therefore, 2040 patients contributed outcome data to this study (table 1). 1126 (55%) participants were men, and the median age of all patients in the validation cohort was 53 years (range 19–87). Median follow-up was 54 months (range 4–105).

Coronary CTA findings are presented in the appendix. During follow-up of the derivation cohort there were 26 confirmed cardiac deaths, 72 confirmed non-cardiac deaths, and 16 deaths from an unknown cause. In the validation cohort, 48 deaths were classified as cardiac, 35 as non-cardiac, and two as of unknown cause (table 1).

**Figure 1:**
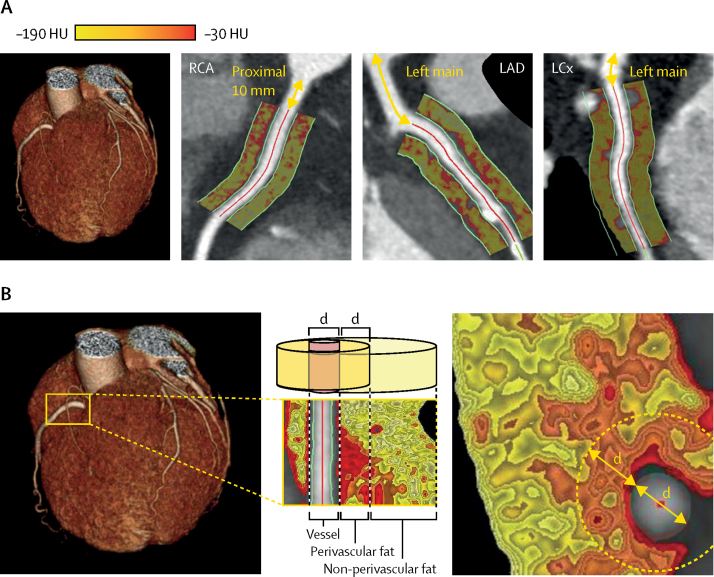
Perivascular FAI analysis around epicardial coronary vessels (A) Perivascular FAI phenotyping of the proximal segments of all three major epicardial coronary vessels, with corresponding FAI colour maps. (B) Example of perivascular FAI phenotyping around the proximal RCA. Perivascular fat was defined as fat within a radial distance equal to the diameter (d) of the vessel. FAI=fat attenuation index. HU=Hounsfield unit. LAD=left anterior descending artery. LCx=left circumflex artery. RCA=right coronary artery.

Perivascular fat attenuation measured around the proximal right coronary artery was associated strongly with perivascular fat attenuation measured around the proximal left anterior descending artery and left circumflex artery (appendix). In both cohorts, high FAI values in the perivascular fat of all three coronary vessels were associated with a significantly higher adjusted risk of all-cause mortality (table 2). However, high FAI values in the perivascular fat were only associated significantly with prospective cardiac mortality risk in the right coronary artery and the left anterior descending artery, not in the left circumflex artery (figure 1, table 2). In view of the statistical collinearity between perivascular FAI measurements around the right coronary artery and the left anterior descending artery, the analysis was restricted to the proximal right coronary artery.

**Table 2:** Risk of all-cause and cardiac mortality with perivascular FAI around the three major coronary arteries

	**All-cause mortality**		**Cardiac mortality**	
	Adjusted HR (95% CI)	p value	Adjusted HR (95% CI)	p value
**RCA**				
Derivation cohort	1·49 (1·20–1·85)	0·0003	2·15 (1·33–3·48)	0·0017
Validation cohort	1·84 (1·45–2·33)	<0·0001	2·06 (1·50–2·83)	<0·0001
**LAD**				
Derivation cohort	1·78 (1·42–2·23)	<0·0001	2·61 (1·60–4·27)	0·0001
Validation cohort	1·77 (1·39–2·27)	<0·0001	1·81 (1·29–2·55)	0·0006
**LCx**				
Derivation cohort	1·37 (1·10–1·70)	0·0045	1·32 (0·83–2·08)	0·24
Validation cohort	1·47 (1·16–1·86)	0·0017	1·29 (0·93–1·79)	0·13

HRs adjusted for age, sex, hypertension, hypercholesterolaemia, diabetes mellitus, smoking status, epicardial adipose tissue volume, extent of coronary artery disease (Duke coronary artery disease index), number of high-risk plaque features, and tube voltage. Adjusted HRs are expressed per 1 SD increment in the perivascular FAI. FAI=fat attenuation index. HR=hazard ratio. LAD=left anterior descending artery. LCx=left circumflex artery. RCA=right coronary artery.

**Figure 2:**
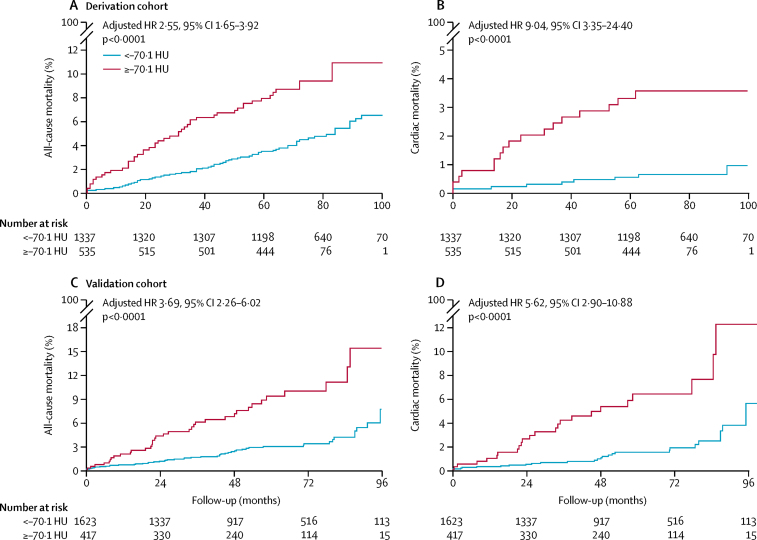
Kaplan-Meier curves of all-cause mortality and cardiac mortality with high versus low perivascular FAI High values for the perivascular FAI were ≥–70·1 HU and low perivascular FAI values were <–70·1 HU. Mortality curves show risk of all-cause mortality in the derivation cohort (A) and validation cohort (C) and cardiac mortality in the derivation cohort (B) and validation cohort (D). HRs are adjusted for age, sex, hypertension, hypercholesterolaemia, diabetes mellitus, smoker status, epicardial adipose tissue volume, tube voltage, extent of coronary artery disease (Duke coronary artery disease index), and number of high-risk plaque features. FAI=fat attenuation index. HR=hazard ratio. HU=Hounsfield unit.

The perivascular FAI was normally distributed around a mean of −75·1 HU (SD 8·6) in the derivation cohort and −77·0 HU (SD 8·5) in the validation cohort. It was associated with neither coronary calcification (measured as CCS) on non-contrast CT (p=0·86) nor local calcium burden in the adjacent vascular segment (p=0·18; appendix). Adjusted fractional polynomial modelling showed a J-shaped relation between the perivascular FAI and the prospective risk of all-cause and cardiac mortality in both cohorts (appendix). Based on this observation, and to generate distinct clinical risk groups, the study population was dichotomised into high versus low perivascular fat attenuation groups based on an optimum cutoff of −70·1 HU (calculated at median follow-up of 72 months [derivation cohort]; specificity 85·0%, sensitivity 67·7%, negative predictive value 99·5%, positive predictive value 5·9%; appendix). In the same cohort, after multivariable adjustment for age, sex, risk factors, tube voltage, modified Duke coronary artery disease index, and number of high-risk plaque features, high perivascular FAI values (≥–70·1 HU *vs* <–70·1 HU) were associated with a higher prospective risk of all-cause mortality (adjusted hazard ratio [HR] 2·55, 95% CI 1·65–3·92; p<0·0001; figure 2A) and cardiac mortality (9·04, 3·35–24·40; p<0·0001; figure 2B). High perivascular FAI values were not associated with non-cardiac mortality (adjusted HR 1·66, 95% CI 0·95–2·90; p=0·07). In the subgroup of patients in whom the extent of coronary calcification was measured with CCS (n=1415 [76%]), the perivascular FAI retained its predictive value for both endpoints after additional adjustment for CCS (adjusted HR 2·03, 95% CI 1·17–3·52; p=0·0122 for all-cause mortality and 12·83, 2·76–59·56; p=0·0011 for cardiac mortality). These findings were confirmed in the validation cohort (specificity 81·6%, sensitivity 52·9%, negative predictive value 98·5%, positive predictive value 7·0%). Perivascular FAI values of −70·1 HU or higher were associated with increased risk of all-cause mortality (adjusted HR 3·69, 95% CI 2·26–6·02; p<0·0001; figure 2C) and cardiac mortality (5·62, 2·90–10·88; p<0·0001; figure 2D).

**Figure 3:**
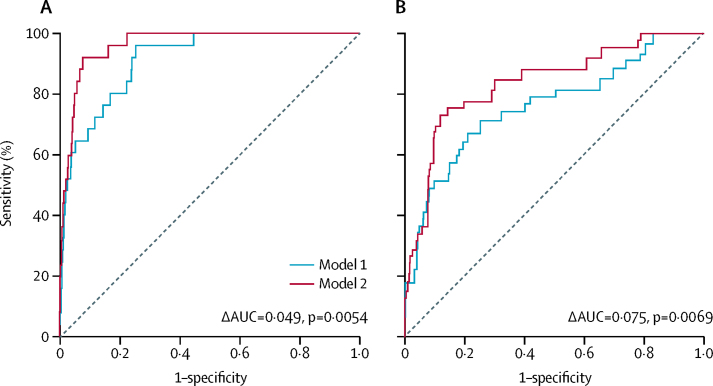
Incremental prognostic value of the perivascular FAI beyond current coronary CTA-based risk stratification Comparison of time-dependent ROC curves (at 6 years) and respective AUC of two nested models for discrimination of cardiac mortality in the (A) derivation and (B) validation cohorts. Model 1 represents the current state-of-the-art in risk assessment and consisted of age, sex, risk factors (hypertension, hypercholesterolaemia, diabetes mellitus, smoker status, epicardial adipose tissue volume), modified Duke coronary artery disease index, and number of high-risk plaque features on coronary CTA. Model 2 incorporates perivascular FAI values (≥–70·1 HU *vs* <–70·1 HU) into model 1. AUC=area under the curve. CTA=computed tomography angiography. FAI=fat attenuation index. HU=Hounsfield unit. ROC=receiver operating characteristic.

Inclusion of high perivascular FAI values (≥–70·1 HU) significantly improved the discriminatory value of a coronary CTA-based risk prediction model that included age, sex, cardiovascular risk factors (hypertension, hypercholesterolaemia, diabetes, smoking, and adipose tissue volume), extent of coronary artery disease (modified Duke coronary artery disease index), and number of high-risk plaque features, for both all-cause and cardiac mortality (figure 3; appendix). For cardiac mortality, in the derivation cohort, with the addition of the perivascular FAI to the risk predication model, the AUC increased from 0·913 (95% CI 0·867–0·958) to 0·962 (0·940–0·983; ΔAUC=0·049; p=0·0054), and in the validation cohort, the AUC increased from 0·763 (95% CI 0·669–0·858) to 0·838 (0·764–0·912; ΔAUC=0·075; p=0·0069; figure 3). Furthermore, the perivascular FAI significantly improved all-cause and cardiac mortality risk stratification in both study cohorts, contributing to substantial net reclassification improvement (table 3), driven primarily by a significant reduction in the estimated risk for non-events, highlighting the high specificity and negative predictive value of the method and proposed cutoff. Among patients without events, the change in estimated risk after perivascular FAI-based risk stratification did not differ between individuals with coronary artery disease versus those without this disease (for all-cause mortality and cardiac mortality, respectively: derivation cohort, p=0·09 and p=0·72; validation cohort, p=0·68 and p=0·40). In the subgroup of individuals in the derivation cohort with available CCS, the perivascular FAI added significant incremental value in risk prediction beyond an enhanced model that included CCS in addition to the previously mentioned risk factors (for all-cause mortality, χ^2^=5·95, p=0·0147 [likelihood ratio test]; for cardiac mortality, χ^2^=10·71, p=0·0011 [likelihood ratio test]).

**Table 3:** Improvement in discrimination and risk reclassification for all-cause and cardiac mortality using the perivascular FAI beyond a current risk prediction model

	**Model performance**		**Discrimination (IDI [95% CI])**	**Risk reclassification**				
	Change in χ^2^	p value*		Events		Non-events		NRI (95% CI)
				Risk up	Risk down	Risk up	Risk down	
**Cardiac mortality**								
Derivation	20·29	<0·0001	0·038 (0·000–0·174)	0·64	0·36	0·17	0·83	0·94 (0·07–1·34)
Validation	25·30	<0·0001	0·032 (0·001–0·090)	0·56	0·44	0·20	0·80	0·72 (0·34–1·07)
**All-cause mortality**								
Derivation	16·54	<0·0001	0·017 (0·003–0·052)	0·48	0·52	0·19	0·81	0·58 (0·35–0·77)
Validation	25·60	<0·0001	0·030 (0·008–0·068)	0·51	0·49	0·21	0·79	0·60 (0·30–0·86)

Perivascular FAI comparison was ≥–70·1 HU *vs* <–70·1 HU. IDI and NRI were calculated at 6 years. Baseline model (current state-of-the-art or model 1): age, sex, hypertension, hypercholesterolaemia, diabetes mellitus, active smoker status, epicardial adipose tissue volume, modified Duke coronary artery disease index (reference: group 1, mild or no disease), and number of high-risk plaque features. New model (model 2): model 1 plus high perivascular FAI values. FAI=fat attenuation index. IDI=integrated discrimination improvement. NRI=net reclassification improvement index. HU=Hounsfield unit.

* Likelihood ratio test.

**Figure 4:**
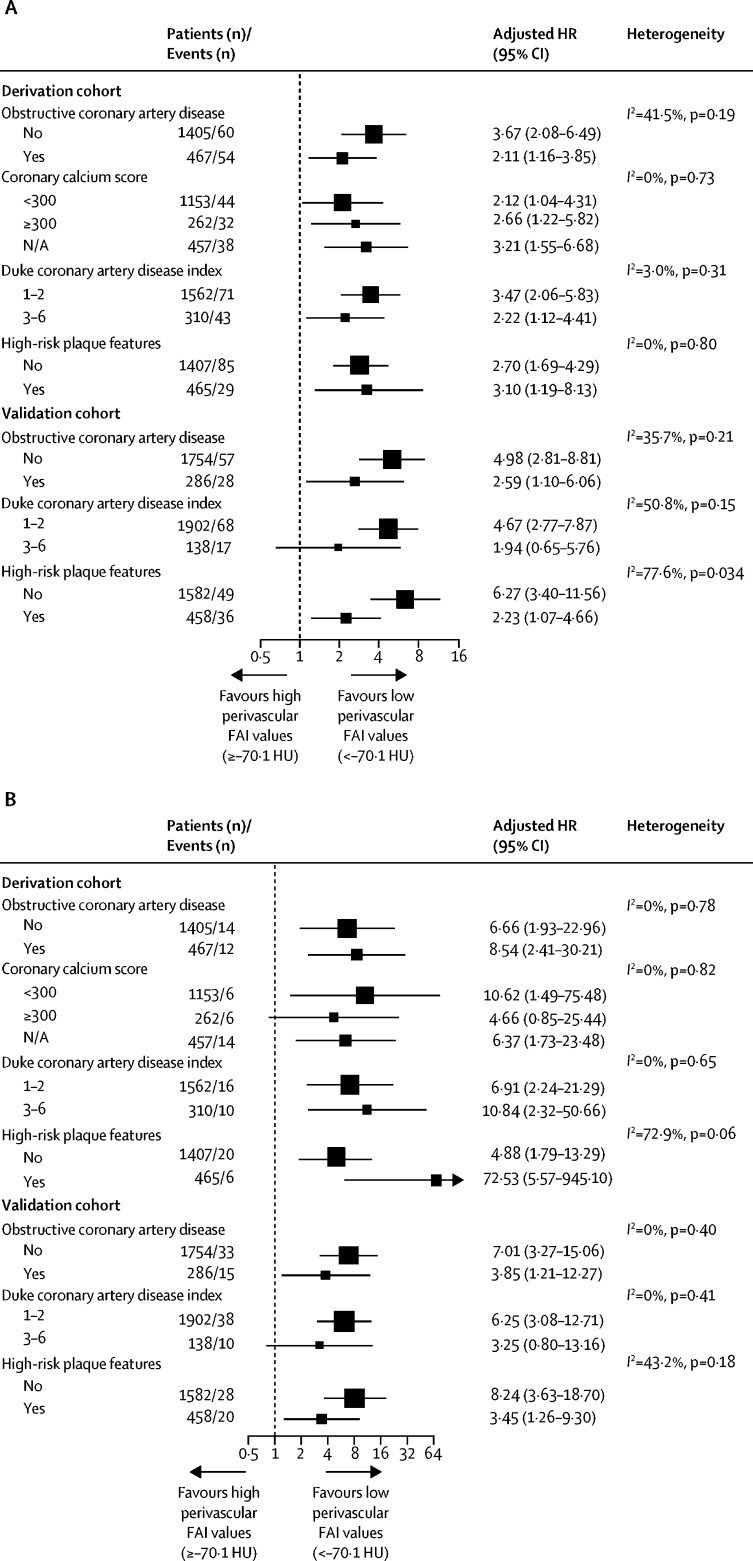
Subgroup analysis of the prognostic value of the perivascular FAI in patients with and without coronary artery disease Plots show adjusted HRs for high versus low perivascular FAI values (≥–70·1 HU *vs* <–70·1 HU) as a prognostic biomarker for (A) all-cause mortality and (B) cardiac mortality in different patient subgroups, with or without cardiac CTA-derived features of coronary artery disease. HRs are adjusted for age, sex, and epicardial adipose tissue volume. CTA=computed tomography angiography. FAI=fat attenuation index. HU=Hounsfield unit. HR=hazard ratio.

In subgroup analyses to assess the performance of the perivascular FAI for both primary and secondary prevention, the perivascular FAI retained its positive association with both all-cause and cardiac mortality in all subgroups, with no significant heterogeneity between subgroups (figure 4). The validation cohort provided information on different ethnic groups and, in a post-hoc analysis, the positive association of high perivascular FAI values with adverse events was consistent across ethnic groups (appendix).

In further subgroup analyses, the association between high perivascular FAI values and mortality risk was independent of indications for coronary CTA referral, presenting symptoms (chest pain; appendix), or recommendations after coronary CTA (appendix). However, among individuals who received a recommendation to initiate treatment with statins or aspirin after coronary CTA, the perivascular FAI (measured before deployment of the new treatment) was no longer predictive of cardiac mortality (adjusted HR 2·85, 95% CI 0·44–18·49; p=0·25). By contrast, among those who did not receive any recommendations for change of management after coronary CTA, the predictive value of high perivascular FAI values for cardiac mortality was retained (adjusted HR 18·71, 95% CI 2·01–174·04; p=0·0101), suggesting that the risk identified by the perivascular FAI could be modifiable with optimum medical therapy.

Availability of information on prospective acute myocardial infarction events in the validation cohort allowed us to further test the prognostic role of theperivascular FAI for this secondary endpoint in a post-hoc analysis. After multivariable adjustment, high perivascular FAI values (≥–70·1 HU *vs* <–70·1 HU) were associated with increased risk of acute myocardial infarction (n=23 reported events; adjusted HR 5·08, 95% CI 1·89–13·61; p=0·0012; appendix), suggesting a link between abnormal perivascular fat attenuation and plaque instability.

## Discussion

The findings of our study show that the imaging biomarker perivascular FAI predicts all-cause and cardiac mortality over and above clinical risk factors and the current state-of-the-art interpretation of coronary CTA. By quantifying the residual inflammatory risk, the perivascular FAI can be used for risk restratification in both primary and secondary prevention, dissociating risk prediction from the strict anatomical severity of coronary stenosis or the degree of myocardial ischaemia. These findings are validated in the CRISP-CT study in two large and different real-life prospective cohorts of patients undergoing clinically indicated coronary CTA in Europe and the USA. Integrating the perivascular FAI into modern coronary CTA interpretation will facilitate identification of individuals at risk of future cardiac death, before structural changes of the coronary wall are visible, and will flag so-called vulnerable patients with inflamed coronary atheroma, who might be candidates for more intensive treatment.

Current risk stratification relies on traditional clinical risk factors, whereas imaging biomarkers such as CCS—measured using CT—are recommended for individuals at low-to-middle risk.^18^ However, coronary calcification represents a non-reversible process that does not regress (or might even increase) in response to appropriate medical treatment (eg, statins), limiting its value in secondary prevention.^19^ On the other hand, inflammation has an important role in both atherogenesis and atherosclerotic plaque rupture leading to acute coronary syndrome.^7^, ^20^ Therefore, non-invasive detection of the residual inflammatory coronary risk could guide more timely deployment of preventive measures in primary care.^21^, ^22^ Moreover, timely detection of inflamed atherosclerotic plaques at risk of rupture would guide the application of secondary prevention measures,^20^, ^21^, ^22^ including novel therapeutic agents that target inflammation.^23^, ^24^, ^25^ After the results of the CANTOS trial,^24^ which confirmed the inflammatory hypothesis of atherosclerosis, a clear unmet need exists to identify patients with inflamed coronary arteries. Indeed, no non-invasive method is readily available to quantify the inflammatory status of coronary vessels or atherosclerotic plaques, and measurement of circulating biomarkers—such as high sensitivity C-reactive protein (hsCRP)—is not specific for coronary inflammation.^26^ Detection of coronary microcalcification and inflammation using PET-CT is promising^27^ but is currently limited by clinical availability, and the prognostic value of this method has not been validated in large cohorts.^28^

Coronary inflammation has been shown to inhibit adipogenesis in perivascular fat,^8^ generating a concentric gradient of adipocyte lipid content around the inflamed coronary artery, with smaller, undifferentiated, and lipid-poor adipocytes closer to the vascular wall. The imaging biomarker under study here (the perivascular FAI) was derived from analysis of standard images obtained by coronary CTA and tracks changes in adipocyte lipid content by mapping the three-dimensional adipose tissue attenuation changes in the perivascular space in a standardised and operator-independent way.^8^ The perivascular FAI changes dynamically in response to local plaque rupture in patients with acute coronary syndrome, and can be used as a so-called thermometer to distinguish potential culprit from non-culprit lesions.^8^ The information captured by the perivascular FAI is independent of coronary calcification (measured by CCS)^8^ or systemic markers of inflammation, such as hsCRP (ρ=–0·11, p=0·25, in an independent cohort of 107 individuals [unpublished data]). Our original findings^8^ have been supported by other studies linking high perivascular fat attenuation with the presence of stable coronary artery disease,^29^ coronary plaque rupture,^30^, ^31^ and spontaneous coronary artery dissection.^31^ However, the value of the perivascular FAI in identifying residual inflammatory risk and predicting cardiac mortality is unknown.

In UK clinical guidelines for management of chest pain,^3^ coronary CTA is recommended as a first-line diagnostic test for assessment of stable coronary artery disease, typical and atypical angina, and non-anginal chest pain with ECG changes. With the implementation of these guidelines, the number of coronary CTA scans undertaken annually in the UK alone could exceed 350 000,^32^ with more than 90% excluding obstructive disease. In the presence of atherosclerotic plaques, coronary CTA identifies specific high-risk plaque features,^15^, ^33^ but the current interpretation lacks the ability to detect small but vulnerable plaques with potential to either rupture or rapidly progress to obstructive disease.^4^ Indeed, evidence suggests that half of acute coronary syndrome events happen without anatomically significant coronary atherosclerosis,^4^ due to rupture of minor but inflamed atherosclerotic plaques.

In our study, we quantified the perivascular FAI in baseline coronary CTA scans from two independent and substantially different prospective cohorts who underwent clinical coronary CTA in Erlangen, Germany (derivation cohort) and Cleveland, OH, USA (validation cohort) and who were followed up for a median of 6 years and 4·5 years, respectively. The clinical indication for coronary CTA was based on local clinical practice, which varied between the two cohorts, as shown by the significantly different clinical and medication profile of the two populations. This diversity provided the opportunity to measure perivascular fat attenuation in patients with a broad range of cardiovascular risk factors and extent of coronary artery disease, scanned using various hardware and during different periods. Traditional risk factors were less effective at predicting risk of mortality in the validation cohort versus the derivation cohort, possibly because of more aggressive pharmacological management of risk factors in the validation cohort. Despite these differences, we showed that a cutoff for the perivascular FAI of −70·1 HU or higher is a strong predictor of all-cause mortality and cardiac mortality in both cohorts, over and above clinical risk factors, extent of coronary artery disease, number of high-risk plaque features, epicardial adipose tissue volume, and coronary calcification (measured by CCS).^10^, ^11^, ^12^ The perivascular FAI also worked well for risk stratification in individuals with low or high CCS, independently of the presence of coronary artery disease. Individuals without coronary artery disease, who comprise most individuals undergoing clinical coronary CTA worldwide, could benefit from early targeted deployment of more aggressive measures of primary prevention. Indeed, we present evidence that the residual inflammatory risk identified by the perivascular FAI might be modifiable by intensive medical treatment, calling for future randomised clinical trials to record this initial observation. Finally, by identifying patients with coronary artery disease who still have unstable atherosclerotic plaques despite optimum treatment, the perivascular FAI can guide future clinical trials assessing targeted treatments with modern anti-inflammatory agents, making their use affordable in modern health-care systems. A clear unmet need also exists to identify patients who remain at risk after acute coronary syndrome, despite standard treatment. Future clinical trials will show whether the perivascular FAI is the long-awaited biomarker that will guide personalised medicine in these patients.

Our study has some limitations. First, the fairly low number of fatal events reported in our study could diminish statistical power for some subgroup analyses, but effect sizes on the primary endpoints were large, which might overcome this shortcoming. Second, although the spatial resolution of CT on a 128-slice scanner could get to a theoretical voxel size of 0·35 mm^2^, this possibility is limited by coronary artery motion.^34^ However, perivascular fat was defined as fat within a radial distance equal to the diameter of the vessel (based on previous histological and biological observations),^8^ which exceeds the spatial resolution of coronary CTA, allowing for a valid assessment of the perivascular space. Third, although our study findings support the use of the perivascular FAI in symptomatic cohorts, its value in asymptomatic cohorts remains unclear. Therefore, the perivascular FAI is not intended to generate new indications for coronary CTA but is proposed as an additional readout in standard coronary CTA done under the current clinical indications. Finally, the goal of our study was to investigate the value of the perivascular FAI around standardised coronary segments as a marker of global residual cardiac risk, and future studies will be needed to assess the prognostic value of lesion-guided perivascular fat attenuation. In this regard, the non-significant association of the perivascular FAI around the left circumflex artery with cardiac mortality could reflect the predominant localisation of culprit coronary lesions in the right coronary artery and left anterior descending artery.^35^ Integration of this method in future clinical trials or application in clinical practice will require development of infrastructure to deliver the perivascular FAI analysis on-site or off-site.

We have shown that the perivascular FAI—a previously validated imaging biomarker of coronary inflammation derived from routine coronary CTA^8^—when measured around the right coronary artery predicts all-cause and cardiac mortality over and above current risk stratification approaches, including measurement of coronary calcium and state-of-the-art assessment of coronary CTA. Integration of the perivascular FAI into standard clinical coronary CTA reporting has the potential to advance this imaging technique from a method that is used to exclude anatomical coronary artery disease into a dynamic cardiac risk-stratification tool, applicable even without coronary plaques or substantial calcification.
