# Supplementary material for: Non-invasive detection of coronary inflammation using computed tomography and prediction of residual cardiovascular risk (the CRISP CT study): a post-hoc analysis of prospective outcome data
Source: Lancet. 2018 Sep 15;392(10151):929–39. doi: 10.1016/S0140-6736(18)31114-0 (PMC6137540; doi:10.1016/S0140-6736(18)31114-0)
Supplement: Supplementary appendix [file mmc1.pdf]

THE LANCET

Supplementary appendix

This appendix formed part of the original submission and has been peer reviewed. We post it as supplied by the authors.

Supplement to: Oikonomou EK, Marwan M, Desai MY, et al. Non-invasive detection of coronary inflammation using computed tomography and prediction of residual cardiovascular risk (the CRISP-CT study): a post-hoc analysis of prospective outcome data. *Lancet* 2018; published online Aug 28. http://dx.doi.org/10.1016/S0140-6736(18)31114-0.

Non-invasive detection of coronary inflammation using computed tomography and prediction of residual cardiovascular risk: a post-hoc analysis of prospective outcome data

Acronym: The **C**ardiovascular **Ris**k **P**rediction using **C**omputed **T**omography **(CRISP-CT)** study

Appendix

Evangelos K. Oikonomou MD[a]†, Mohamed Marwan MD[b]†, Prof. Milind Y. Desai MD[c]†, Jennifer Mancio MD[a], Alaa Alashi MD[c], Erika Hutt Centeno MD[c], Sheena Thomas BSc[a], Laura Herdman BSc[a], Christos P. Kotanidis MD[a], Katharine E. Thomas MRCP[a], Brian P. Griffin MD[c], Scott D. Flamm MD[c], Alexios S. Antonopoulos MD[a], Cheerag Shirodaria MD[d], Nikant Sabharwal DM[d], Prof. John Deanfield FRCP[e], Prof. Stefan Neubauer MD[a,f,g], Jemma C. Hopewell PhD[h], Prof. Keith M. Channon MD[a,f,g], Prof. Stephan Achenbach MD[b], Prof. Charalambos Antoniades PhD[a,f,g]*

[a] Division of Cardiovascular Medicine, Radcliffe Department of Medicine, University of Oxford, UK.
[b] Department of Cardiology, Friedrich-Alexander-Universität Erlangen-Nürnberg, Erlangen, Germany.
[c] Cleveland Clinic Heart and Vascular Institute, Cleveland, USA.
[d] Cardiology Department, Oxford University Hospitals NHS Foundation Trust, Oxford, UK.
[e] UCL Institute of Cardiovascular Science, London, UK.
[f] Oxford Centre of Research Excellence, British Heart Foundation, UK.
[g] Oxford Biomedical Research Centre, National Institute of Health Research, UK.
[h] Clinical Trial Service Unit, Nuffield Department of Population Health, University of Oxford, UK.
†Contributed equally to the study.

Supplemental Figures: **11**, Supplemental Tables: **3**

Corresponding author:
Prof. Charalambos Antoniades MD PhD,
Professor of Cardiovascular Medicine
Division of Cardiovascular Medicine, University of Oxford,
John Radcliffe Hospital, Oxford OX3 9DU, United Kingdom,
Tel: +44-1865-228340, Fax: +44-1865-740352, e-mail: antoniad@well.ox.ac.uk

Supplemental Methods

Data collection: In both cohorts, outcome data were assembled through search of medical records, and querying of local/national databases by local investigators not involved in subsequent image/data analysis. Clinical data and demographics were recorded prospectively in the electronic medical records at the time of the initial clinical encounter and manually extracted for the current study. The beginning of follow-up was considered as the date of the CCTA scan. The primary endpoints of the study were the occurrence of all-cause and cardiac mortality. In the Cleveland (validation) cohort, additional data on myocardial infarction events during follow-up (ST-segment elevation or non-ST segment elevation) were retrieved through search of electronic health records.

Endpoint definitions: For the purposes of this study, the investigators agreed to follow the guidelines of the ACC/AHA (American College of Cardiology/American Heart Association)[1] and the Academic Research Consortium for definition of the cause of death.[2] Cardiac mortality was defined as any death due to proximate cardiac causes (e.g. myocardial infarction, low-output heart failure, fatal arrhythmia). Deaths fulfilling the criteria of sudden cardiac death were also included in this group.[1,2] Any death not covered by the previous definition, such as death caused by malignancy, accident, infection, sepsis, renal failure, suicide or other non-cardiac vascular causes such as stroke or pulmonary embolism was classified as non-cardiac.[1,2] Deaths where information on the exact cause could not be collected with certainty were classified as "deaths of unknown cause" at the discretion of the local site investigators.

Event adjudication: Adjudication of events and the exact cause of death was performed internally within each site by local study investigators not involved in subsequent image or statistical analysis, through chart review, inspection of the death certificate and/or telephone follow-up and/or verification with a family member. For the classification of the cause of death, study investigators followed the guidelines of the ACC/AHA[1] and the Academic Research Consortium,[2] as described above. All information was sent for statistical analysis to an independent team based at the University of Oxford, United Kingdom, which performed all analyses blindly.

Risk factors: In regression models, hypertension was defined based on the presence of a documented diagnosis or treatment with an antihypertensive regimen, according to the relevant clinical guidelines.[3] Similar criteria were applied for the definition of hypercholesterolemia and diabetes mellitus.[4,5]

Pre-test probability of coronary artery disease (CAD): The pre-test probability of CAD in the included study population was calculated by using the updated formula of the CAD Consortium, using all available clinical variables (age, sex, hypertension, hypercholesterolaemia, diabetes mellitus, smoking, atypical/typical angina) and assuming a high-prevalence geographical setting.[6]

Coronary Artery Disease (CAD) assessment: Included scans were reviewed by at least two independent researchers. Obstructive CAD was defined as the presence of at least one coronary stenosis ≥50% on coronary computed tomography angiography (CCTA). Mild, moderate and severe coronary stenoses were defined as lesions causing luminal stenosis 25-49%, 50-69% and ≥70%, respectively.[7] The extent of CAD on CCTA was assessed by the Modifed Duke Prognostic CAD Index, as previously described.[8] Based on this classification system, the study population was split in six independent groups, as follows:
Group 1: <50% stenosis
Group 2: ≥2 mild stenoses with proximal CAD in 1 artery or 1 moderate stenosis
Group 3: 2 moderate stenoses or 1 severe stenosis
Group 4: 3 moderate stenoses, 2 severe stenoses, or severe stenosis in the proximal left anterior descending (LAD) artery.
Group 5: 3 severe stenoses or 2 severe stenoses in the proximal LAD
Group 6: ≥50% stenosis in left main coronary artery.

High-risk plaque features on CCTA: The presence of high-risk plaque features on CCTA was defined by two independent researchers (EKO, JM) based on the presence of at least one of the following features, as previous described.[9] Low attenuation plaque (LAP) was defined in the presence of low CT attenuation in a non-calcified plaque. Operators placed 3 random region-of-interest measurements (approximately 0·5-1·0 mm^2) in the non-calcified low CT attenuation portion of the plaque. If the mean CT number within these 3 regions of interest was <30 HU (Hounsfield Units), the patient was identified as having LAP. Spotty calcification was defined as the presence of calcified plaque with a diameter <3 mm in any direction, length (extent in the longitudinal direction of the vessel) of the calcium less than 1·5 times the vessel diameter and width (extent of the calcification perpendicular to the longitudinal direction of the vessel) of the calcification <2/3 of the vessel diameter. Positive remodelling was assessed visually in multi-planar reformatted images reconstructed in long axis and short axis view of the vessel. The remodeling index (RI) was calculated by dividing the cross-sectional lesion diameter by the diameter of a proximal, reference segment and a threshold of 1·1 was used to define positive remodeling. Napkin ring sign was defined as a ring-like peripheral higher attenuation of the non-calcified portion of the coronary plaque.[9]

Coronary computed tomography angiography (CCTA) protocol

Erlangen (derivation) cohort: CCTA scans were performed in a 2 x 64-slice scanner (Definition Flash, Siemens Healthcare, Forchheim, Germany) (n=1482, 79·2%), with the remainder using either a 64-slice (Siemens Sensation 64, Siemens Healthcare, Forchheim, Germany) (n=339, 18·1%) or 2 x 128-slice scanner (n=71, 2·7%) (Somatom Definition Flash, Siemens Healthcare, Forchheim, Germany). Oral medication with 100 mg atenolol was administered one hour before CT if heart rate was > 60 beats per minute with additional 5 mg doses of metoprolol intravenously up to a maximum dose of 30 mg, if the heart rate remained above 60 beats per minute once the patient was positioned on the CT table. Patients also received 0·8 mg of nitroglycerine sublingually immediately before CCTA and iodinated contrast (Omnipaque 350, Schering AG, Berlin, Germany) was administered at flow rate of 5-6 ml/s.

Cleveland (validation) cohort: The majority of the CCTA scans (n=1777, 87·1%) were performed in a 256-slice Brilliance iCT scanner (Philips Medical Systems, Best, The Netherlands), with the remainder using a 2 x 128-slice Definition Flash scanner (Siemens Healthcare, Erlangen, Germany) (n=221, 10·8%) and a 2 x 192-slice Somatom Force CT scanner (Siemens Healthcare, Forchheim, Germany) (n=42, 2·1%). In patients with heart rate > 60 beats/minute, 5 mg of intravenous metoprolol (with incremental 5 mg doses up to a maximum dose of 30 mg) or intravenous diltiazem (5 mg increments up to 20 mg maximum), if the heart rate remained above 60 beats per minute once the patient was positioned on the CT table. Patients also received 0·3 mg of nitroglycerin sublingually immediately before CCTA and iodinated contrast (Omnipaque 350, General Electric, Milwaukee, USA) was administered at flow rate of 5-6 ml/s.

CCTA post-processing and image analysis

The reconstructed images were transferred to a processing workstation (Aquarius Workstation® V.4.4.11-13, TeraRecon Inc., Foster City, CA, USA). All scans were reviewed based on their quality. Scans were excluded from inclusion in the study in the presence of severe artefacts, missing slices, coronary abnormalities or if done at tube voltage other than 100 or 120 kVp, where FAI_{PVAT} has been validated.[10,11] To adjust for differences in attenuation between scans done at different tube voltages, the perivascular adipose tissue fat attenuation index (FAI) for scans performed at 100kVp was divided by a conversion factor of 1·11485 to be comparable to scans performed at 120kVp, as previously validated.[10,11] Tube voltage was also included as a factor in all relevant multiple regression models. Scans performed at different tube voltage settings (as shown in Figure 1 in the main manuscript-Study Flowchart) were excluded, since the FAI_{PVAT} analysis methodology has not been validated yet at these settings.[9] Scans performed at 140, 110, 90 and 70 kVp were only present in the Cleveland cohort, accounting for just 2·1% (48/2246) of the total number of scans. Similarly, scans performed at 80 kVp accounted for 0·7% (14/1993) and 0.6% (14/2246) of the total number of CCTA scans in the Erlangen, and Cleveland cohorts respectively. In the latter, all 80 kVp scans were

performed in 2016 suggesting a possible change in clinical practice, which could introduce a bias compared to older cases from within the same cohort.

Proximal, 40 mm-long segments of all major three epicardial coronary vessels (right coronary artery[RCA], left anterior descending artery [LAD] and left circumflex artery [LCx]) were tracked using a dedicated CCTA analysis software (Aquarius Workstation® V.4.4.11-13, TeraRecon Inc., Foster City, CA, USA). To avoid the effects of the aortic wall, the most proximal 10 mm of the RCA were excluded and analysis was performed in the proximal 10-50 mm of the vessel, as previously described.[11] In the LAD and LCx, analysis was performed in the proximal 40 mm of each vessel. If the obtuse marginal was clearly a more dominant vessel than the LCx, analysis was performed extending into the former vessel. The left main coronary artery was not analysed because of its variable length and anatomy and because it may be absent. Following identification of the segment of interest, the lumen as well as the inner and outer wall border were tracked in an automated manner with additional manual optimization. Previously validated Hounsfield Unit (HU) thresholds were applied for characterization of vascular wall components (65 to 260 HU for fibrous plaque and >465 HU for calcification).[12] The calcified burden of a coronary segment were calculated by dividing the total voxel volume of the calcified (>465 HU)[12] component of the coronary wall by the total volume of the respective vessel segment. PVAT was defined as the adipose tissue located within a radial distance from the outer vessel wall equal to the diameter of the respective vessel.[11] Voxel attenuation histograms were plotted and the weighted average attenuation of all voxels between -30 to -190 HU (thresholds used for the definition of AT)[13,14] within the PVAT volume was used for the calculation of FAI_{PVAT}, following adjustment for tube voltage.[10,11] The total epicardial adipose tissue (EAT) volume was assessed in a semi-automated manner by tracking the contour of the pericardium from the level of the pulmonary artery bifurcation to the apex of the heart at the most caudal end. Four researchers blinded to patient demographics and outcomes worked independently to analyze the vascular wall and perivascular / epicardial adipose tissue. Of note, the intra-observer and inter-observer agreement for FAI_{PVAT} was excellent (intra-class correlation coefficient: 0·987 [P<0·0001] and 0·980 [P<0·0001] respectively). The mean attenuation of the aortic root was measured at the level of the origin of the left main coronary artery by positioning a circular region of interest in the aortic lumen.

Coronary calcium score (CCS): In the derivation cohort only, CCS was quantified by the Agatston method on non-contrast cardiac CT scans using commercially available software (Aquarius Workstation® V.4.4.11-13, TeraRecon Inc., Foster City, CA, USA), in those patients with an indication for CCS assessment.[15-17]

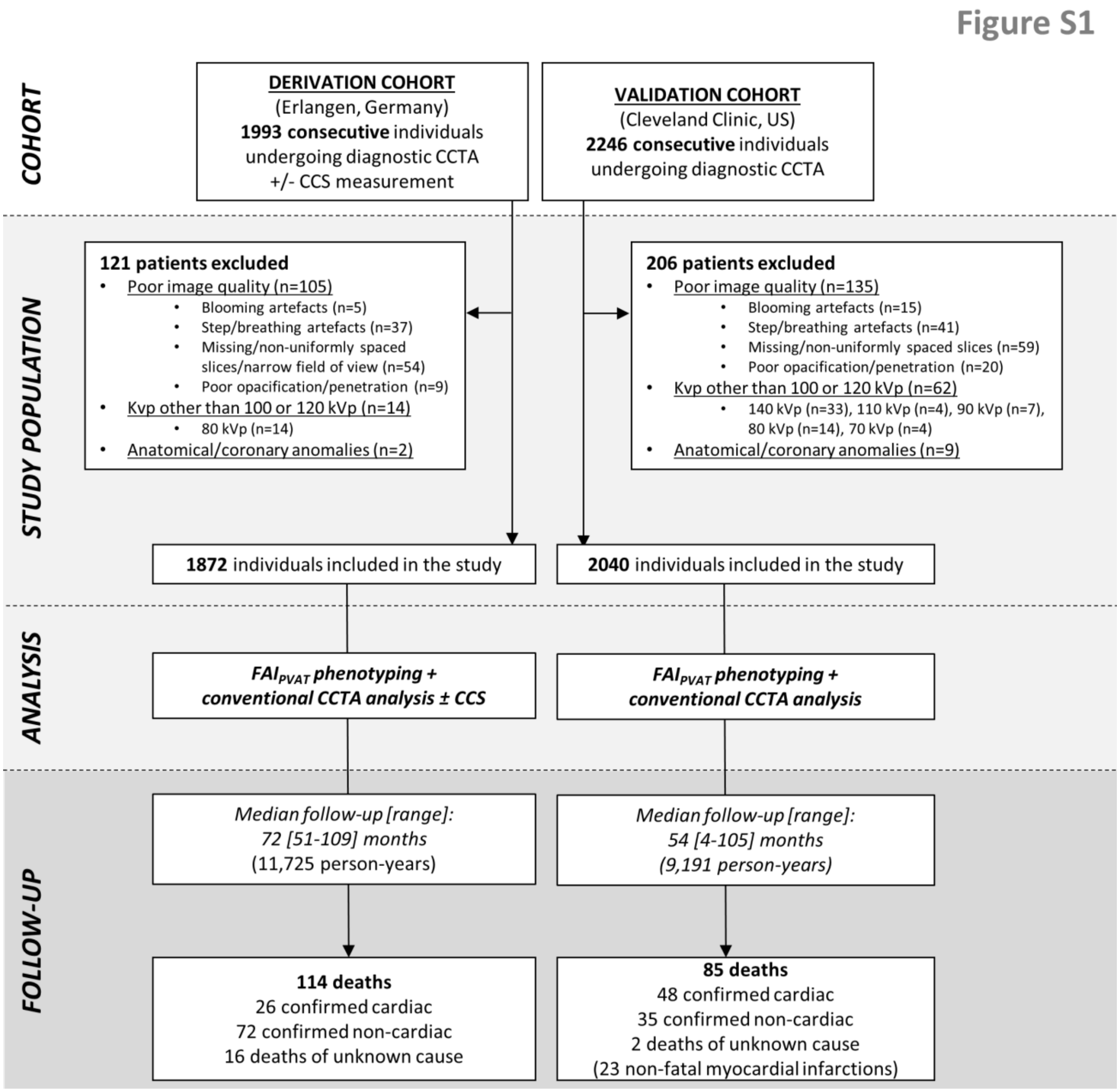

Figure S1. Study design and flowchart.
CCS: Agatston coronary calcium score; CCTA: coronary computed tomography angiography; FAI_{PVAT}: fat attenuation index of the perivascular adipose tissue.

Figure S2

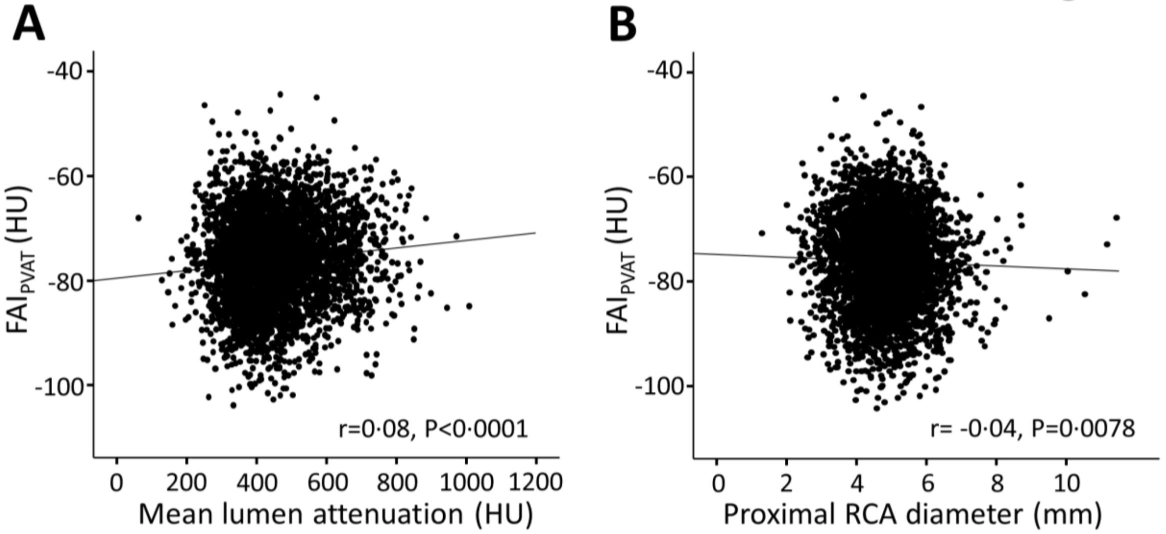

C **Association of FAI_{PVAT} with technical/anatomical parameters.**

**Multiple linear regression model [Dependent variable: FAI_{PVAT} (HU)]**				
**Independent variables**	**Unstandardised coefficient**	**Standardised coefficient**	**P value**	**R^2 of the model**
Lumen attenuation (HU)	0·007	0·10	<0·0001	
RCA diameter (mm)	-0·253	-0·026	0·10	0·05
Tube voltage (120 vs 100 kVp)	-5·315	-0·278	<0·0001	

FAI_{PVAT}: fat attenuation index of the perivascular adipose tissue; HU: Hounsfield Units; RCA: right coronary artery.

Figure S2. Association of FAI_{PVAT} with technical and anatomical parameters. (A) Bivariate association between FAI_{PVAT} and mean lumen attenuation in the aortic root. **(B)** Bivariate association between FAI_{PVAT} and mean diameter of the proximal RCA. **(C)** Multiple linear regression model with FAI_{PVAT} as the dependent variable and mean lumen attenuation, vessel diameter and tube voltage as the independent predictors. FAI_{PVAT}: fat attenuation index of the perivascular adipose tissue; HU; Hounsfield Units; RCA: right coronary artery.

Figure S3

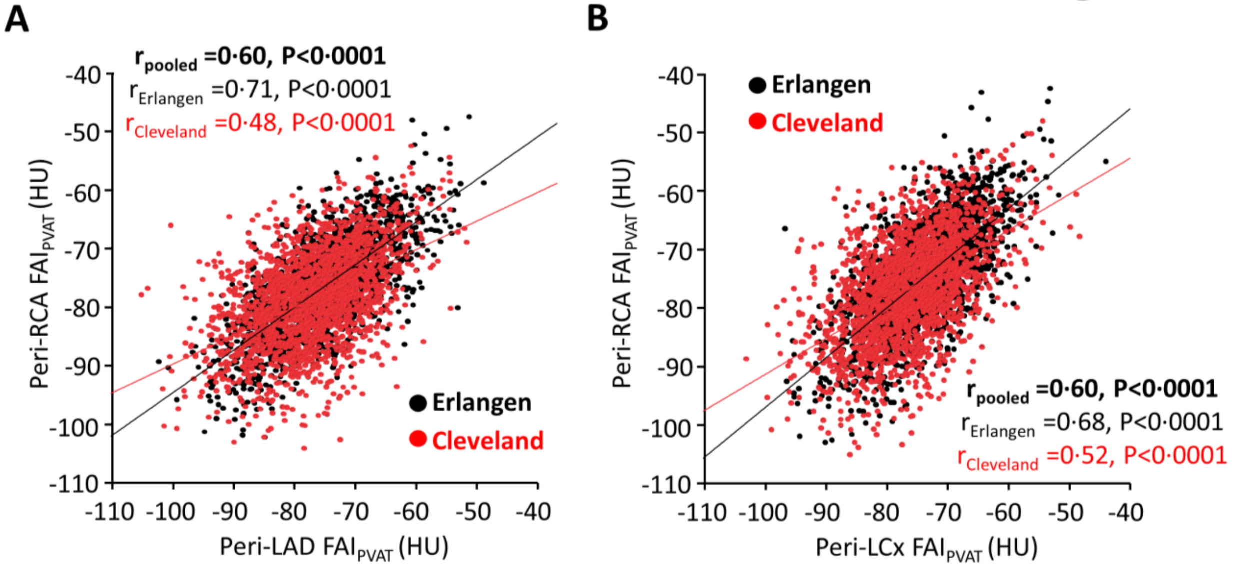

Figure S3. Bivariate associations of FAI_{PVAT} measured around the three major epicardial coronary vessels. Correlation between FAI_{PVAT} values measured around the RCA and LAD/LCx. FAI_{PVAT}: fat attenuation index of the perivascular adipose tissue; HU: Hounsfield Units; LAD: left anterior descending artery; LCx: left circumflex; RCA: right coronary artery.

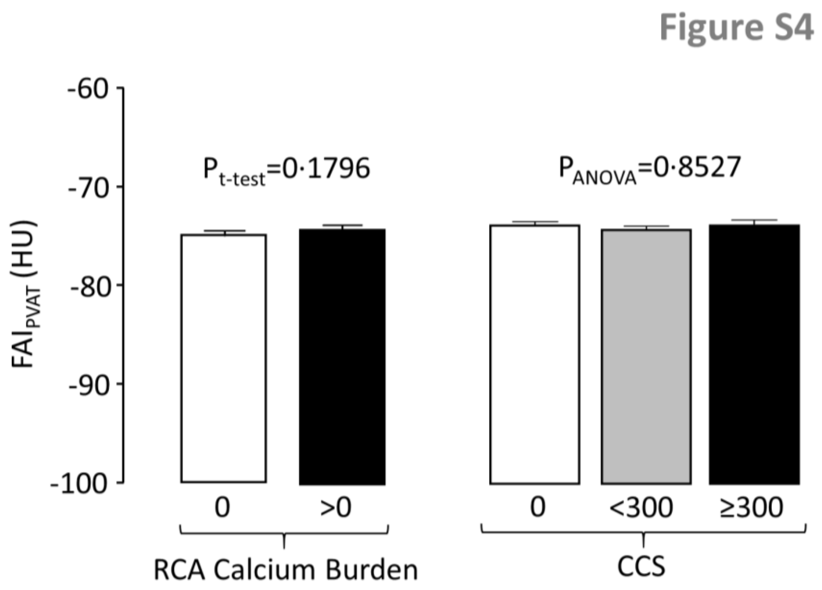

Figure S4. Association of FAI$_{PVAT}$ with local and global coronary calcium. In the Erlangen cohort, FAI$_{PVAT}$ was independent of coronary calcium, measured either in the whole coronary tree on non-contrast images (Agatston coronary calcium score, CCS) or in the adjacent vascular segment on contrast CCTA images (calcium burden of the RCA). ANOVA: analysis of variance; CCS: Agatston coronary calcium score; CCTA: coronary computed tomography angiography; FAI$_{PVAT}$: fat attenuation index of the perivascular adipose tissue; RCA: right coronary artery.

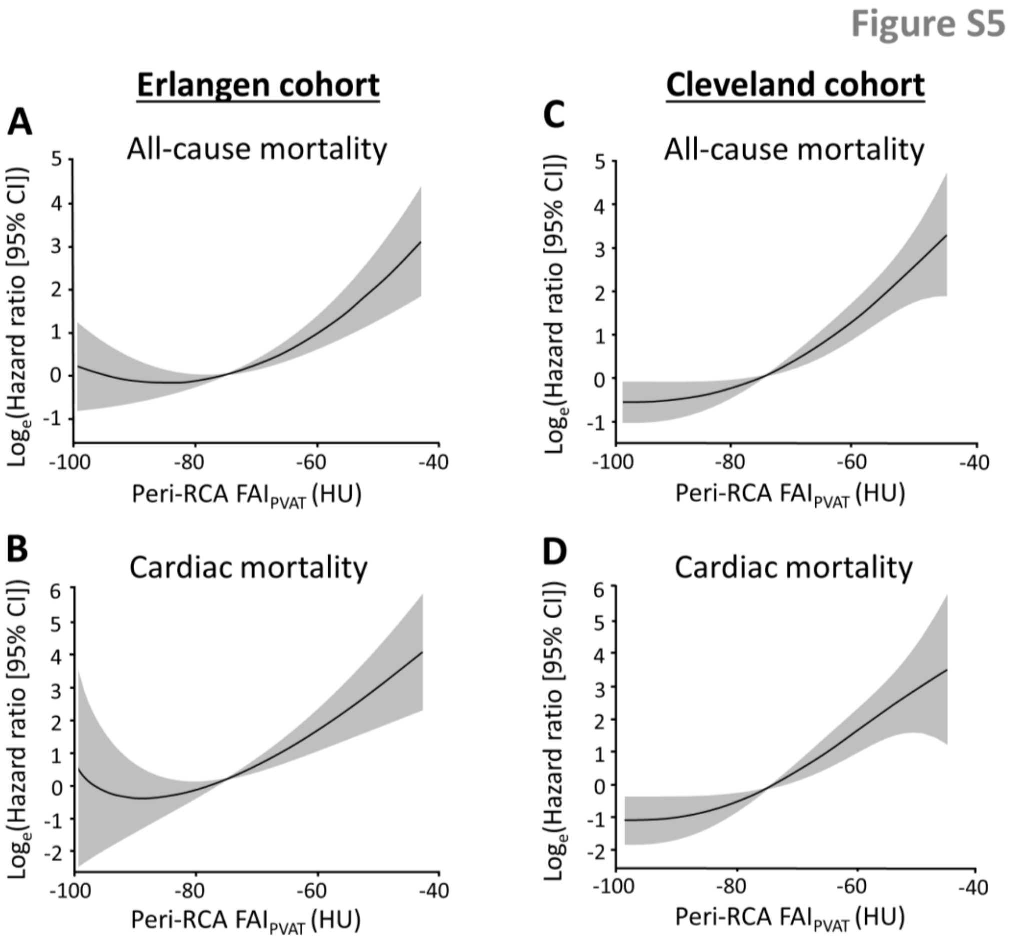

Figure S5. Fractional polynomial models to assess non-linear associations between FAI$_{PVAT}$ and mortality risk. (A, B) Best fitting fractional polynomials for FAI$_{PVAT}$ as a predictor of all-cause and cardiac mortality, in the Erlangen (A, B) and Cleveland cohorts (C, D), respectively. All models demonstrate a J-shaped association between FAI$_{PVAT}$ at baseline and prospective mortality risk. Models are adjusted for age, sex, tube voltage, and epicardial adipose tissue volume. CI: confidence interval; FAI$_{PVAT}$: Fat Attenuation Index of Perivascular Adipose Tissue; HU: Hounsfield Units.

Figure S6

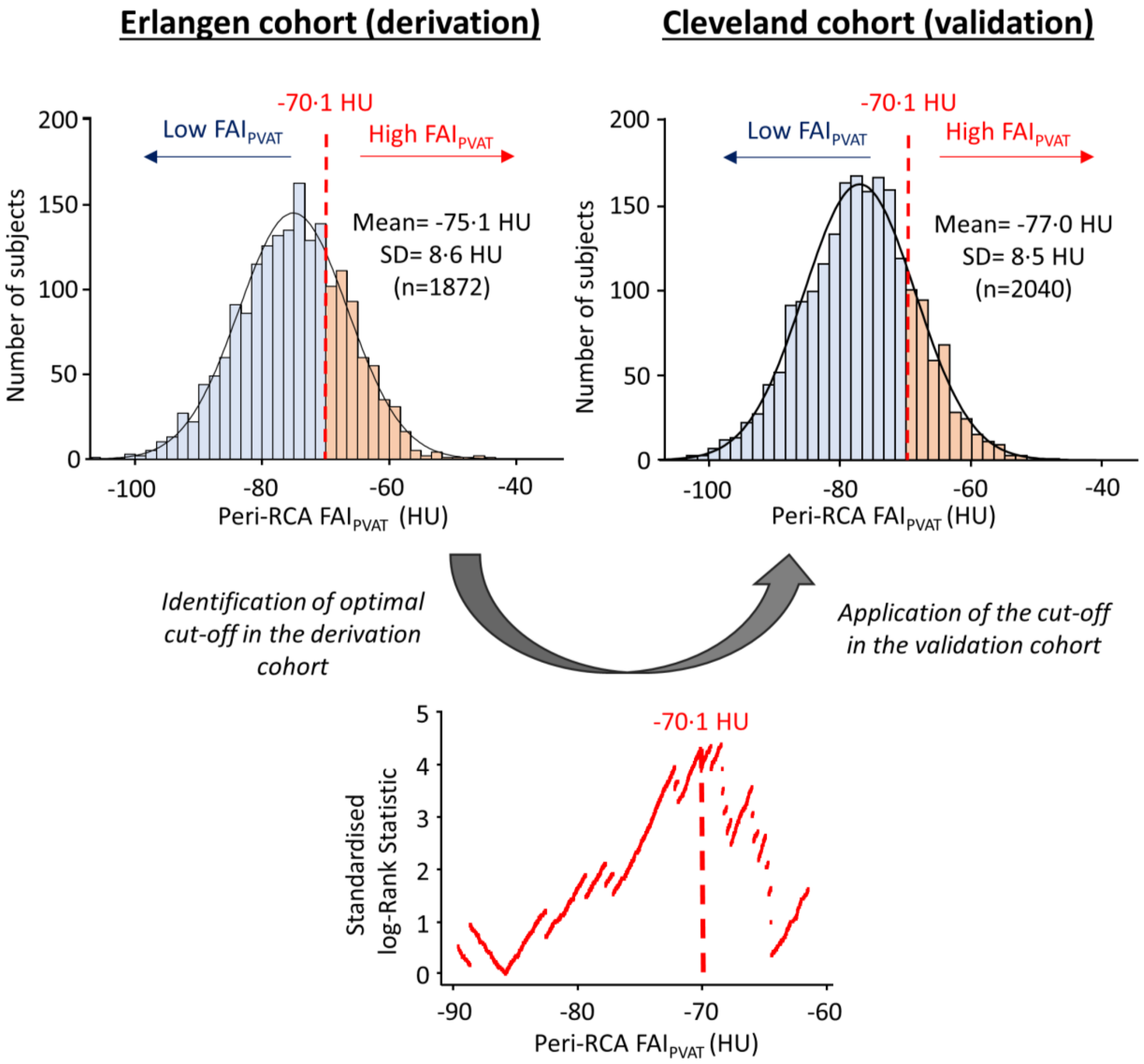

Figure S6. Identifying an optimal cutoff for FAI_{PVAT} as a prognostic biomarker. An optimal cutoff was selected in the Erlangen (derivation) cohort, by identifying the FAI_{PVAT} value (around the reference segment of the proximal right coronary artery) that maximized the Youden's J index (sum of sensitivity and specificity) in time-dependent receiver operating characteristic curves for the primary endpoint of cardiac mortality at t_0=6 years (72 months, corresponding to the median follow-up interval in this cohort). The derived cutoff was subsequently applied in both the derivation (Erlangen) cohort and the Cleveland (validate) cohort in order to validate its prognostic value for cardiac mortality. FAI_{PVAT}: fat attenuation index of the perivascular adipose tissue; HU: Hounsfield Units; RCA: right coronary artery.

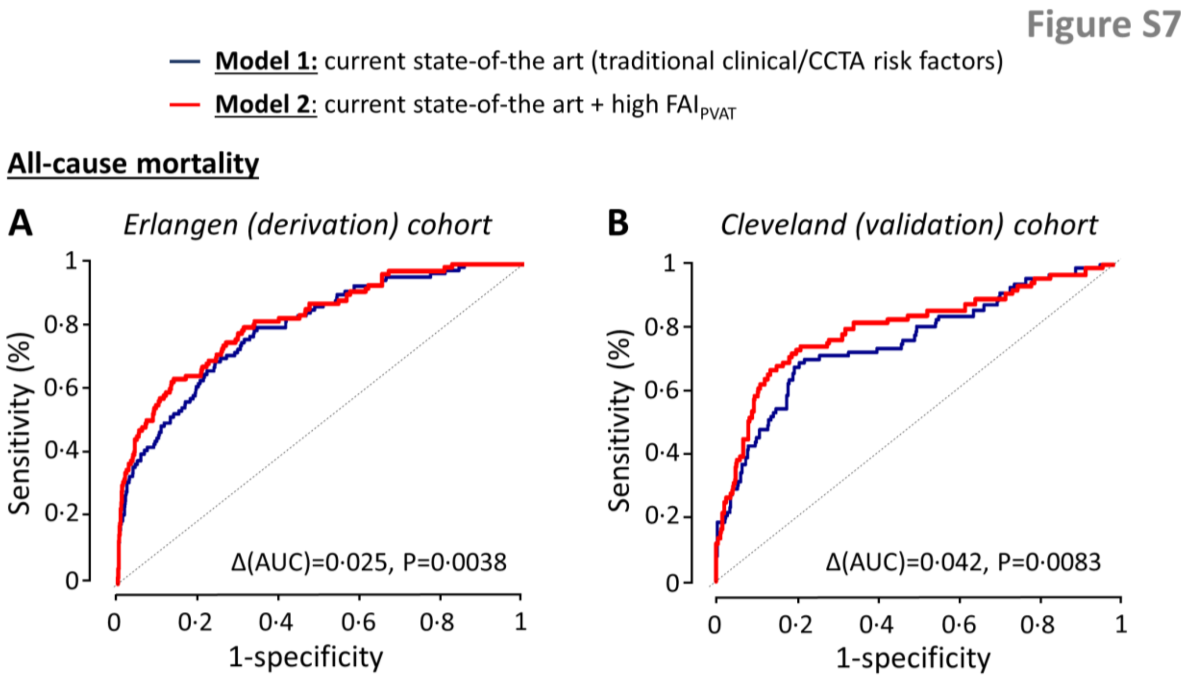

Figure S7. Incremental prognostic value of FAI_{PVAT} for all-cause mortality beyond current CCTA-based risk stratification. Model 1 represents the current-state-of-the-art in risk assessment and consisted of age, sex, risk factors (hypertension, hypercholesterolaemia, diabetes mellitus, smoking status, epicardial adipose tissue volume), modified Duke Prognostic CAD index and number of high-risk plaque features on CCTA, while Model 2 was constructed by incorporating high FAI_{PVAT} (≥ vs < -70·1 HU) into Model 1. (A-B) Comparison of time-dependent ROC curves (at t_0=6 years) and respective AUC of the two nested Models 1 & 2 (before, and after the addition of FAI_{PVAT}) for discrimination of all-cause mortality in the Erlangen (A) and Cleveland cohorts (B). In the Erlangen cohort (A), the AUC increased from 0·800 (95% CI: 0·752-0·848) to 0·825 (95% CI: 0·778-0·872) ($P_{\Delta AUC}$=0.0038), whereas in the Cleveland cohort (B), the AUC increased from 0·755 (95% CI: 0·686-0·823) to 0·797 (95% CI: 0·731-0·863) ($P_{\Delta AUC}$=0.0083). AUC: area under the curve; CAD: coronary artery disease; CCTA: coronary computed tomography angiography; CI: confidence interval; FAI_{PVAT}: fat attenuation index of the perivascular adipose tissue; ROC: receiver operating characteristic.

Figure S8

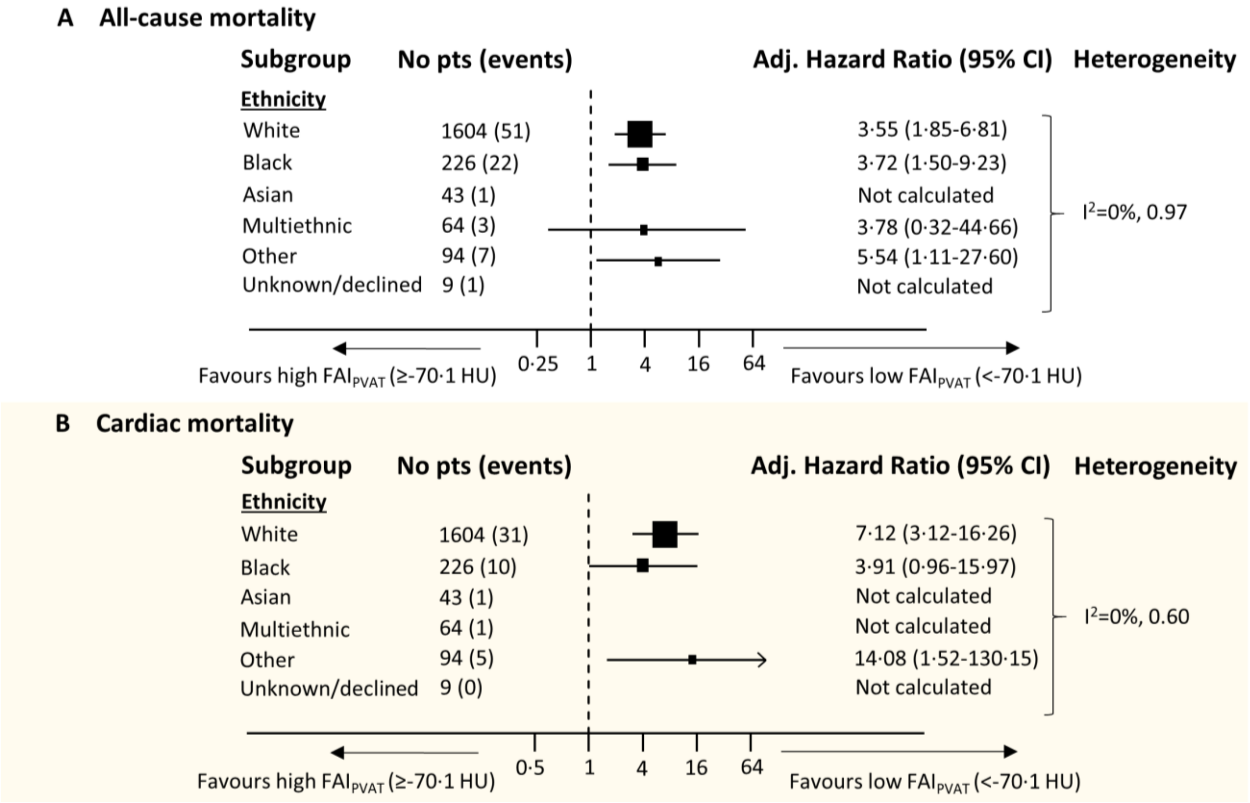

Figure S8. Subgroup analysis of the predictive value of FAI_{PVAT} by ethnicity. In a post-hoc analysis of data collected from the Cleveland cohort, the positive association between FAI_{PVAT} and all-cause (A) or cardiac mortality (B) was found to be independent of the ethnic background of the included study population (no between-group heterogeneity). Models adjusted for age, sex and epicardial adipose tissue volume. CI: confidence interval; FAI_{PVAT}: fat attenuation index of perivascular adipose tissue; HU: Hounsfield Units; pts: patients.

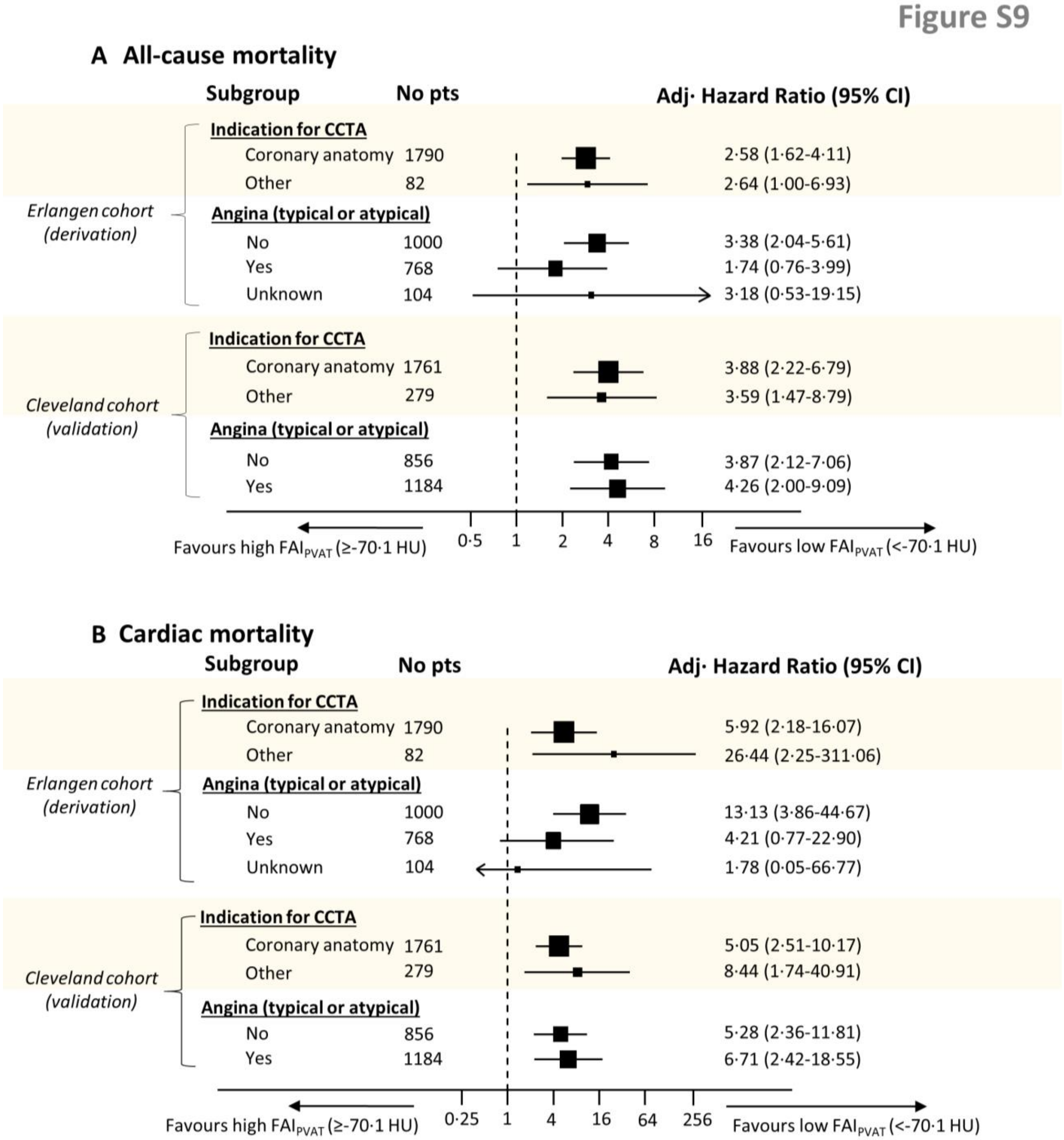

Figure S9. Subgroup analysis of the predictive value of FAI_{PVAT} by indication for CCTA and presenting symptoms. To explore whether different indications for CCTA or different presenting symptoms may affect the predictive value of FAI_{PVAT} for all-cause (A) and cardiac mortality (B), subgroup analysis was performed using adjusted Cox regression models (adjusted for age, sex and epicardial adipose tissue volume) stratified by: i. the indications for CCTA referral (grouped in coronary versus non-coronary indications) and ii. presenting symptoms (presence versus absence of chest pain/angina). FAI_{PVAT} retained its positive association with the prospective risk of both all-cause and cardiac mortality in both cohorts, with no evidence of heterogeneity between different indications for CCTA or presenting symptoms ($I^2<20\%$ and $P>0{\cdot}05$ for all between-subgroup comparisons). This supports the predictive value of FAI_{PVAT} as a risk biomarker in a wide range of patients referred for CCTA. CCTA: coronary computed tomography angiography; CI: confidence interval; FAI_{PVAT}: fat attenuation index of perivascular adipose tissue; HU: Hounsfield Units; pts: patients.

Figure S10

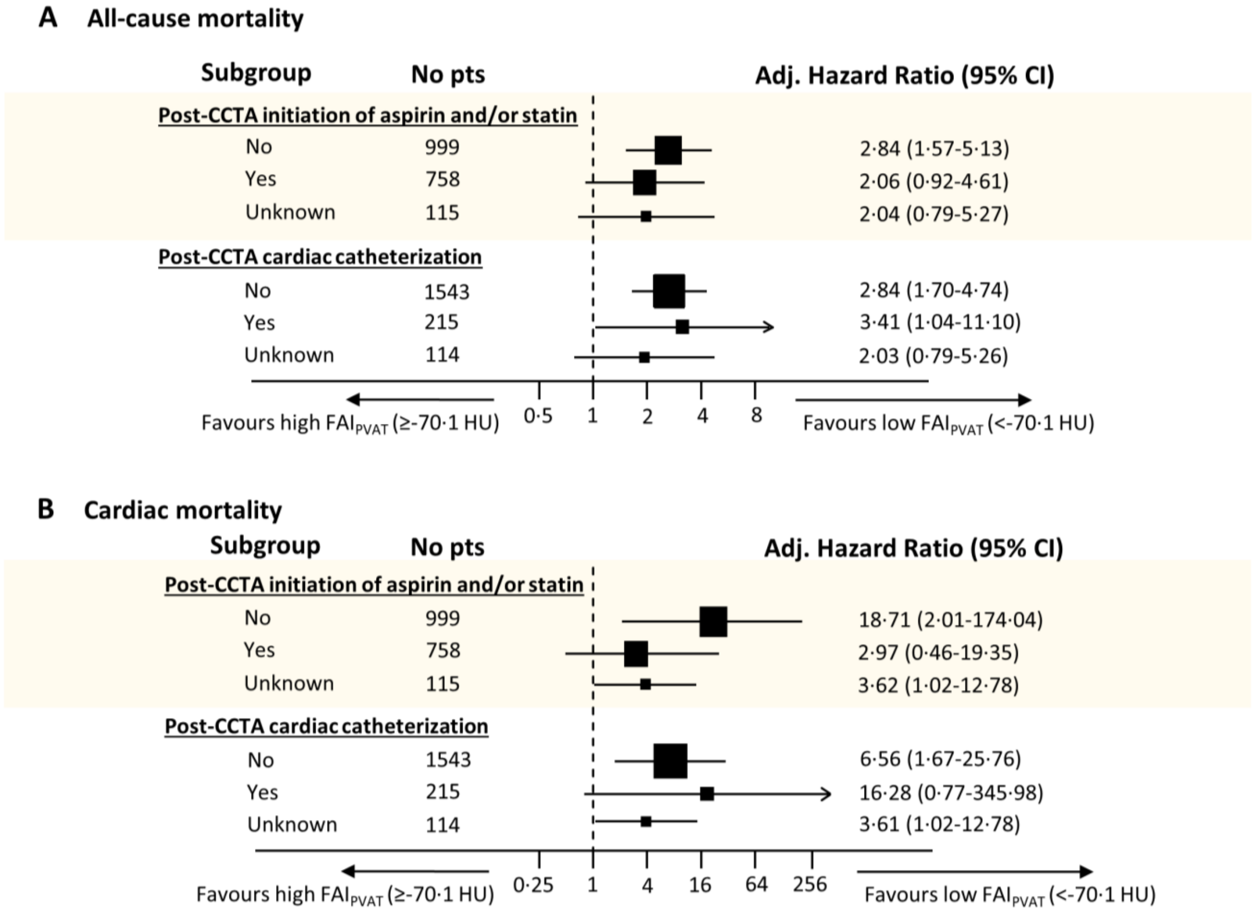

Figure S10. Subgroup analysis of the predictive value of FAI$_{PVAT}$ based on post-CCTA changes in medical management. To explore whether different recommendations post-CCTA may have affected the predictive value of FAI$_{PVAT}$ for all-cause **(A)** and cardiac mortality **(B)**, subgroup analysis was performed in the derivation (Erlangen) cohort using adjusted Cox regression models (adjusted for age, sex and epicardial adipose tissue volume) stratified by the following post-CCTA recommendations: i. treatment with statin and/or aspirin; and ii. referral for cardiac catheterization. FAI$_{PVAT}$ retained its positive association with the prospective risk of both all-cause **(A)** and cardiac mortality **(B)** in all subgroups. Nevertheless, it is worth highlighting that FAI$_{PVAT}$ was strongly associated with cardiac mortality events in patients that did not receive recommendations for treatment with statins or aspirin, while the association in the group that did receive such recommendations was non-significant. This may suggest a role for FAI$_{PVAT}$ in guiding the deployment of secondary prevention measures in patients that do not qualify for medical treatment based on conventional CCTA analysis and highlights a significant room for improvement in current clinical practice. CCTA: coronary computed tomography angiography; CI: confidence interval; FAI$_{PVAT}$: fat attenuation index of perivascular adipose tissue; HU: Hounsfield Units; pts: patients.

Figure S11

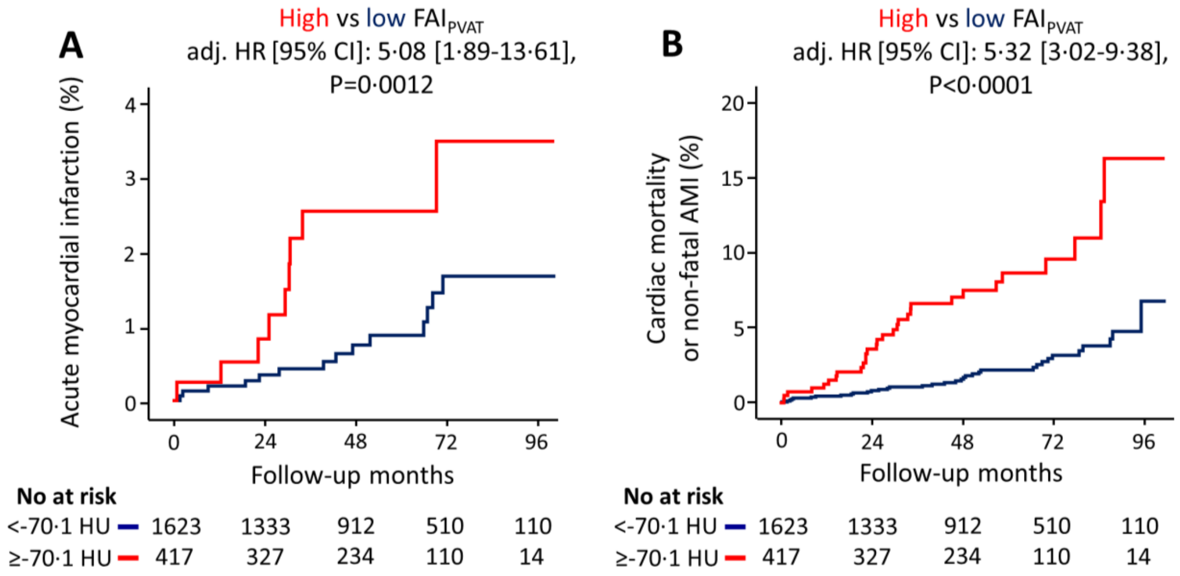

Figure S11. Predictive value of FAI_{PVAT} for acute myocardial infarction events. In the validation (Cleveland) cohort, additional prospective follow-up information on the secondary outcome of acute myocardial infarction (AMI) events, revealed a significant higher risk of AMI in patients with high versus low FAI_{PVAT} values at baseline (≥ versus < -70·1HU). Following multivariable adjustment for age, sex, risk factors (hypertension, hypercholesterolaemia, diabetes mellitus, smoker status, epicardial adipose tissue volume), tube voltage, extent of CAD (assessed by the modified Duke Prognostic CAD Index), and number of high-risk plaque features on CCTA, those in the high FAI_{PVAT} group had a five-fold higher risk of suffering an AMI event (A, n=23 events) or a major adverse cardiac event (MACE) (B, n=65 events), defined as the composite endpoint of non-fatal myocardial infarction and cardiac mortality. AMI: acute myocardial infarction; CAD: Coronary Artery Disease; CCTA: coronary computed tomography angiography; CI: Confidence Interval; FAI_{PVAT}: Fat Attenuation Index of Perivascular Adipose Tissue; HR: Hazard Ratio; HU: Hounsfield units.

Supplemental Tables

Table S1. Presenting symptoms and indications for CCTA in the study population.

	Erlangen Cohort (Derivation)	Cleveland Clinic (Validation)
**Total number included, n (%)**	1872 (100)	2040 (100)
**Pre-test likelihood of CAD (%)***	30·4 [15·4-50·4]	21·5 [9·3-38·7]
**Indications for CCTA referral, n (%)**		
Assessment for CAD/coronary anatomy	1790 (95·6)	1761 (86·4)
Other non-coronary indications	82 (4·4)	279 (13·6)
**Symptoms prior to CCTA, n (valid %)**		
Chest pain	768 (43·4)	1184 (58·0)
Dyspnoea	193 (10·8)	452 (22·2)
Palpitations	240 (13·5)	225 (11·0)

CAD: coronary artery disease; CCTA: coronary computed tomography angiography; IQR: interquartile range. Valid % refers to the study population with known symptoms, after excluding individuals with undocumented symptoms. *median [IQR]

Table S2. Break-down of CCTA findings in the derivation and validation cohorts.

	Erlangen cohort (Derivation)	Cleveland Clinic cohort (Validation)
**Subjects included in the study, n (%)**	**1872 (100)**	**2040 (100)**
**Total CCS score, n(%)**		
0	526 (28·1)	-
1-99	444 (23·7)	-
100-299	183 (9·8)	-
≥300	262 (14·0)	-
Not performed	457 (24·4)	100·0
**Coronary Artery Disease: maximal stenosis, n (%)**		
None to mild (<30%)	673 (36·0)	1033 (50·6)
Mild (30-50%)	732 (39·0)	721 (35·4)
Moderate (50-70%)	226 (12·1)	196 (9·6)
Severe (≥70%)	241 (12·9)	90 (4·4)
**Extent of CAD (Modified Duke Prognostic CAD Index), n (%)**		
**Group 1**: <50% stenosis	1044 (55·8)	1690 (82·8)
**Group 2**: ≥2 Mild Stenoses with Proximal CAD in 1 Artery or 1 Moderate Stenosis	518 (27·7)	212 (10·4)
**Group 3**: 2 Moderate Stenoses or 1 Severe Stenosis	66 (3·5)	100 (4·9)
**Group 4**: 3 Moderate Stenoses, 2 Severe Stenoses, or Severe Stenosis in the Proximal LAD	152 (8·1)	9 (0·4)
**Group 5**: 3 Severe Stenoses or 2 Severe Stenoses in the Proximal LAD	18 (1·0)	14 (0·7)
**Group 6**: ≥50% stenosis in left main coronary artery	74 (3·9)	15 (0·7)
**High-risk plaque features, n (%)**		
Any	465 (24·8)	458 (22·5)
Spotty calcification	417 (22·3)	407 (20·0)
Low-attenuation plaque	84 (4·5)	64 (3·1)
Positive remodeling	72 (3·9)	126 (6·2)
Napkin-ring sign	51 (2·7)	55 (2·7)

CAD: coronary artery disease; CCTA: coronary computed tomography angiography; CCS: Agatston coronary calcium score; LAD: left anterior descending artery.

Table S3. Sensitivity and specificity of different FAI_{PVAT} cut-offs for prediction of cardiac mortality in the derivation (Erlangen) cohort.

FAI_{PVAT} cutoff (HU)	Specificity	Sensitivity
**-66**	92·7	40·1
**-68**	89·0	44·0
**-70**	85·1	63·7
-70·1	**85·0**	**67·7**
**-72**	79·4	71·7
**-74**	70·8	75·6
**-76**	60·4	75·6
**-78**	52·2	83·4
**-80**	41·8	87·3
**-82**	32·8	87·3
**-84**	24·3	91·3
**-86**	17·3	91·3

Specificity and sensitivity presented at different cutoffs of FAI_{PVAT} (per increments of 2 HU in the range of -66 to -86 HU, including the optimal cut-off of -70·1 HU as identified by the Youden's J index (maximal sum of sensitivity and specificity) in the derivation cohort. All presented at t_0=6 years (median follow-up). FAI_{PVAT}: fat attenuation index of the perivascular adipose tissue; HU: Hounsfield Units.

Supplemental References

1. Hicks KA, Tcheng JE, Bozkurt B, et al. 2014 ACC/AHA Key Data Elements and Definitions for Cardiovascular Endpoint Events in Clinical Trials: A Report of the American College of Cardiology/American Heart Association Task Force on Clinical Data Standards (Writing Committee to Develop Cardiovascular Endpoints Data Standards). *J Am Coll Cardiol* 2015; **66**(4): 403-69.
2. Cutlip DE, Windecker S, Mehran R, et al. Clinical end points in coronary stent trials: a case for standardized definitions. *Circulation* 2007; **115**(17): 2344-51.
3. James PA, Oparil S, Carter BL, et al. 2014 evidence-based guideline for the management of high blood pressure in adults: report from the panel members appointed to the Eighth Joint National Committee (JNC 8). *JAMA* 2014; **311**(5): 507-20.
4. American Diabetes A. Diagnosis and classification of diabetes mellitus. *Diabetes Care* 2014; **37 Suppl 1**: S81-90.
5. Stone NJ, Robinson JG, Lichtenstein AH, et al. 2013 ACC/AHA guideline on the treatment of blood cholesterol to reduce atherosclerotic cardiovascular risk in adults: a report of the American College of Cardiology/American Heart Association Task Force on Practice Guidelines. *J Am Coll Cardiol* 2014; **63**(25 Pt B): 2889-934.
6. Genders TS, Steyerberg EW, Hunink MG, et al. Prediction model to estimate presence of coronary artery disease: retrospective pooled analysis of existing cohorts. *BMJ* 2012; **344**: e3485.
7. Cury RC, Abbara S, Achenbach S, et al. Coronary Artery Disease - Reporting and Data System (CAD-RADS): An Expert Consensus Document of SCCT, ACR and NASCI: Endorsed by the ACC. *JACC Cardiovasc Imaging* 2016; **9**(9): 1099-113.
8. Min JK, Shaw LJ, Devereux RB, et al. Prognostic value of multidetector coronary computed tomographic angiography for prediction of all-cause mortality. *J Am Coll Cardiol* 2007; **50**(12): 1161-70.
9. Puchner SB, Liu T, Mayrhofer T, et al. High-risk plaque detected on coronary CT angiography predicts acute coronary syndromes independent of significant stenosis in acute chest pain: results from the ROMICAT-II trial. *J Am Coll Cardiol* 2014; **64**(7): 684-92.
10. Okayama S, Soeda T, Takami Y, et al. The influence of effective energy on computed tomography number depends on tissue characteristics in monoenergetic cardiac imaging. *Radiol Res Pract* 2012; **2012**: 150980.
11. Antonopoulos AS, Sanna F, Sabharwal N, et al. Detecting human coronary inflammation by imaging perivascular fat. *Sci Transl Med* 2017; **9**(398).
12. Obaid DR, Calvert PA, Gopalan D, et al. Atherosclerotic plaque composition and classification identified by coronary computed tomography: assessment of computed tomography-generated plaque maps compared with virtual histology intravascular ultrasound and histology. *Circ Cardiovasc Imaging* 2013; **6**(5): 655-64.
13. Tamarappoo B, Dey D, Shmilovich H, et al. Increased pericardial fat volume measured from noncontrast CT predicts myocardial ischemia by SPECT. *JACC Cardiovasc Imaging* 2010; **3**(11): 1104-12.
14. Hell MM, Achenbach S, Schuhbaeck A, Klinghammer L, May MS, Marwan M. CT-based analysis of pericoronary adipose tissue density: Relation to cardiovascular risk factors and epicardial adipose tissue volume. *J Cardiovasc Comput Tomogr* 2016; **10**(1): 52-60.
15. Stone NJ, Robinson JG, Lichtenstein AH, et al. 2013 ACC/AHA guideline on the treatment of blood cholesterol to reduce atherosclerotic cardiovascular risk in adults: a report of the American College of Cardiology/American Heart Association Task Force on Practice Guidelines. *Circulation* 2014; **129**(25 Suppl 2): S1-45.
16. Greenland P, Alpert JS, Beller GA, et al. 2010 ACCF/AHA guideline for assessment of cardiovascular risk in asymptomatic adults: executive summary: a report of the American College of Cardiology Foundation/American Heart Association Task Force on Practice Guidelines. *Circulation* 2010; **122**(25): 2748-64.
17. Goff DC, Jr., Lloyd-Jones DM, Bennett G, et al. 2013 ACC/AHA guideline on the assessment of cardiovascular risk: a report of the American College of Cardiology/American Heart Association Task Force on Practice Guidelines. *J Am Coll Cardiol* 2014; **63**(25 Pt B): 2935-59.
